# Metatranscriptomic profiling reveals pathogen and host response signatures of pediatric acute sinusitis and upper respiratory infection

**DOI:** 10.1186/s13073-025-01447-3

**Published:** 2025-03-17

**Authors:** Andrew C. Doxey, Nooran Abu Mazen, Max Homm, Vivian Chu, Manjot Hunjan, Briallen Lobb, Sojin Lee, Marcia Kurs-Lasky, John V. Williams, William MacDonald, Monika Johnson, Jeremy A. Hirota, Nader Shaikh

**Affiliations:** 1 Department of Biology, University of Waterloo, 200 University Avenue West, Waterloo, ON N2L 3G1 Canada; 2 Waterloo Centre for Microbial Research, University of Waterloo, 200 University Avenue West, Waterloo, ON N2L 3G1 Canada; 3 Cheriton School of Computer Science, University of Waterloo, 200 University Avenue West, Waterloo, ON N2L 3G1 Canada; 4 Firestone Institute for Respiratory Health, St. Joseph’s Hospital, 50 Charlton Avenue East, Hamilton, ON L8N 4A6 Canada; 5 Department of Medicine, University of British Columbia, 2775 Laurel Street Vancouver, British Columbia, V5Z 1M9 Canada; 6 Faculty of Health Sciences, Department of Medicine, McMaster University, 1200 Main Street West, ON Hamilton, L8N 3Z5 Canada; 7 Division of General Academic Pediatrics, School of Medicine, University of Pittsburgh, UPMC Children’s Hospital of Pittsburgh, 4401 Penn Avenue, Pittsburgh, PA 15224-1334 USA; 8 Division of Infectious Diseases, School of Medicine, University of Pittsburgh, 1218 Scaife Hall 3550 Terrace Street, Pittsburgh, PA USA

**Keywords:** Acute sinusitis, Host response, Upper respiratory infections, Pediatrics, Metatranscriptomics, RNA-seq

## Abstract

**Background:**

Acute sinusitis (AS) is a frequent cause of antibiotic prescriptions in children. Distinguishing bacterial AS from common viral upper respiratory infections (URIs) is crucial to prevent unnecessary antibiotic use but is challenging with current diagnostic methods. Despite its speed and cost, untargeted RNA sequencing of clinical samples from children with suspected AS has the potential to overcome several limitations of other methods. In addition, RNA-seq may reveal novel host-response biomarkers for development of future diagnostic assays that distinguish bacterial from viral infections. There are however no available RNA-seq datasets of pediatric AS that provide a comprehensive view of both pathogen etiology and host immune response.

**Methods:**

Here, we performed untargeted RNA-seq (metatranscriptomics) of nasopharyngeal samples from 221 children with AS and performed a comprehensive analysis of pathogen etiology and the impact of bacterial and viral infections on host immune responses. Accuracy of RNA-seq-based pathogen detection was evaluated by comparison with culture tests for three common bacterial pathogens and qRT-PCR tests for 12 respiratory viruses. Host gene expression patterns were explored to identify potential host responses that distinguish bacterial from viral infections.

**Results:**

RNA-seq-based pathogen detection showed high concordance with culture or qRT-PCR, showing 87%/81% sensitivity (sens) / specificity (spec) for detecting three AS-associated bacterial pathogens, and 86%/92% (sens/spec) for detecting 12 URI-associated viruses, respectively. RNA-seq also detected an additional 22 pathogens not tested for clinically and identified plausible pathogens in 11/19 (58%) of cases where no organism was detected by culture or qRT-PCR. We reconstructed genomes of 196 viruses across the samples including novel strains of coronaviruses, respiratory syncytial virus, and enterovirus D68, which provide useful genomic data for ongoing pathogen surveillance programs.

By analyzing host gene expression, we identified host-response signatures that differentiate bacterial and viral infections, revealing hundreds of candidate gene biomarkers for future diagnostic assays.

**Conclusions:**

Our study provides a one-of-kind dataset that profiles the interplay between pathogen infection and host responses in pediatric AS and URI. It reveals bacterial and viral-specific host responses that could enable new diagnostic approaches and demonstrates the potential of untargeted RNA-seq in diagnostic analysis of AS and URI.

**Supplementary Information:**

The online version contains supplementary material available at 10.1186/s13073-025-01447-3.

## Background

Clinically, acute bacterial sinusitis (hereinafter referred to as acute sinusitis) is diagnosed in children when bacterial superinfection of inflamed mucosa secondary to an upper respiratory tract viral infection (URTI) is suspected [1, 2]. It is one of the most common diagnoses in pediatric primary care settings in the USA with 5 million antibiotic prescriptions written annually [3]. However, because symptoms of acute sinusitis and an uncomplicated URTI overlap considerably, some children diagnosed and treated for acute sinusitis do not have a bacterial infection [2, 3]. The diagnosis is especially challenging because the symptoms may be less specific in young children [2]. Overtreatment of infections such as sinusitis is thought to be a major contributor to the rise in antimicrobial resistance (AMR), which remains an ongoing threat to public health [1].

Bacterial pathogens most frequently isolated from the sinuses of children with acute sinusitis include *Haemophilus influenzae* (HFLU), *Streptococcus pneumoniae* (SPN), and *Moraxella catarrhalis* (MCAT) [2, 4]. Upper respiratory tract infections are often associated with viruses such as influenza virus (INF), respiratory syncytial virus (RSV), coronavirus (COV), adenovirus (ADV), human rhinovirus (HRV), human metapneumovirus (MPV), enterovirus (EV), and parainfluenza virus (PIV) [5]. Symptoms of a viral upper respiratory tract infection can be difficult to distinguish from symptoms of acute bacterial sinusitis [5].

Recently, it has been suggested that one way to distinguish between bacterial and viral infections would be to obtain samples from the middle turbinate or nasopharynx of children with suspected sinusitis and to test these samples (using culture or qRT-PCR) for the three bacterial pathogens that frequently cause acute sinusitis [6]. Using the presence of bacterial pathogens in the nasopharynx to determine which children benefit from antibiotics, which was the paradigm shift suggested by the above manuscript [6], does not require claiming that these pathogens are necessarily causing an infection or that they are present in the sinuses. This shift was necessary in the view of the authors of the aforementioned work because establishing whether bacterial sinusitis is truly present is neither ethical nor practical; the latter could only be accomplished by aspirating every single sinus and determining whether pathogens are present and whether they are causing inflammation.

With the remarkable reduction in the cost of high-throughput sequencing technologies, sequencing has emerged as an appealing strategy for the detection and taxonomic characterization of microorganisms in clinical samples from patients and has potential to overcome several limitations of currently available methods such as culture or qRT-PCR [7, 8]. High-throughput sequencing of RNA transcripts derived from all organisms (bacterial, viral, host, etc.) in a patient sample (metatranscriptomics [9]) allows for a broad, untargeted approach to detect common, uncommon, and novel pathogens. Pathogen detection by high-throughput RNA or DNA sequencing is showing promise in a growing number of infectious disease applications including pneumonia [10, 11], COVID-19 [12], meningitis [13], and febrile illness [14], and has been effective in identifying potential pathogens causing infection, including cases where no pathogen was detected using qRT-PCR or culture.

In addition, a significant benefit of metatranscriptomic sequencing is that it captures both pathogen-derived as well as host-derived RNA, which facilitates both pathogen detection as well as analysis of host gene expression patterns (host response profiling). Whereas sequence-based pathogen detection relies on detecting sequences of known pathogens, host-response profiling may quantify the expression level of biomarkers that indicate active host immune response to infection in a pathogen-agnostic manner. For example, Wesolowska-Andersen et al. used dual RNA-seq to examine host-virus interactions in asthmatic children, and found that patients with high viral read counts were associated with host-response gene expression indicating immune cell infiltration, cilia downregulation, and dampening of the type 2 inflammatory response [15]. Also using metatranscriptomics, Zhang et al. found that host responses to upper respiratory viral infection can impact host-microbiome interactions such as antibiotic resistance gene expression that play a role in secondary bacterial infections [16]. Thus, information on host-response may help not only to distinguish active infections from colonization but also uncover potential host biomarkers of infectious disease progression and severity. Several previous studies have also used RNA-seq or microarray techniques to identify and quantify biomarkers that differentiate between viral and bacterial respiratory infections [17–22]. Using 104 host-response genes identified using microarray analysis of blood samples, Tsalik et al. developed separate bacterial and viral infection classifiers that had a combined accuracy of 87% [17]. Host-response profiling from blood samples has also formed the basis of commercially available systems (e.g., MeMed BV®). If host-response profiles from a nasopharyngeal (NP) sample can similarly be used to differentiate bacterial from non-bacterial sinusitis infections, this could contribute to the development of biomarker assays that inform clinical decision making regarding the use of antibiotics.

In this work, to examine the ability of metatranscriptomics to uncover microbiological and clinically relevant information, we performed metatranscriptomic analysis of NP swabs from 221 children with clinically diagnosed acute sinusitis who were a subset of children enrolled in a previously described clinical trial [6]. Through RNA-seq analysis of NP swab samples, we performed metatranscriptomic pathogen detection and assessed its ability to reproduce culture and qRT-PCR results for 3 bacteria and 12 viruses. We then reconstructed partial to complete genomes of 196 viruses. Finally, we performed host-response profiling and identified gene expression signatures of bacterial and viral infection, which correlated significantly with pathogen load. Our work shows the potential of metatranscriptomics for improving diagnosis of sinusitis and upper respiratory tract infections.

## Methods

### Study design and description of the cohort

Between February 2016 and April 2022, 510 children 2 to 11 years of age (inclusive) with clinically diagnosed acute sinusitis as defined by the American Academy of Pediatrics were enrolled in a randomized multicenter double-blind trial (ClinicalTrials.gov number, NCT02554383). As described previously [6], an initial Pediatric Rhinosinusitis Symptom Scale (PRSS) score was required for inclusion. Children with persistent presentation (nasal and/or cough for 11 to 30 days without improvement) as well as children with worsening presentation (nasal or cough or fever in day 6 to 10 who appeared to be recovering from a viral URI) were included. The main exclusion criteria were severe disease and systemic antibiotic use within 15 days [6]. Children were recruited from 6 outpatient centers. Children were randomly assigned to receive 10 days of amoxicillin-clavulanate or matching placebo. A total of 204 patients did not have a NP sample collected, or their sample was not preserved in RNA buffer and was excluded. Of the remaining 306 patients’ samples, 61 were not sequenced due to low RNA yield. Although 245 samples underwent RNA sequencing, batch 1 was prepared with a different kit/protocol and when analyzed displayed a strong batch effect and was thus removed, leaving 221 patients. The previously reported primary outcome, symptom severity, was assessed by having parents complete the PRSS electronically every evening on days 2 to 11 [6].

In addition to the above cohort, we also included 9 children as control samples. These children were asymptomatic household contacts of index cases enrolled in a separate study (STUDY20070001: INSPIRE—Infection iN houSehold contacts of Patients with covId-19: The Role of Epigenetics) who were recently diagnosed with COVID-19 based on a positive qRT-PCR test. Index cases were recruited from pediatric outpatient clinics, urgent care facilities, and emergency departments, as well as through the Pitt + Me online research recruitment platform. Household contacts were eligible if they tested negative for COVID-19 and the following symptoms were absent: (1) fever plus cough or difficulty breathing; or (2) fever or cough plus loss of taste or smell. Enrollment occurred between April 2021 and July 2023.

### Sample collection

We collected NP swabs from all 221 children at study entry. As previously described [23], the tip of the swab was cut, placed in a cryovial with DNA/RNA shield (Zymo, R1100), and transported on ice to the lab; this cryovial was used for RNA-seq. For the nine control samples, participants received sterile flocked swabs along with a preservation tube containing 3 mL of buffer (DNA/RNA Shield, Zymo Research, Irvine, CA, USA) for self- or parental swabbing. The collected specimens were sent back to the research laboratory via medical courier without the use of ice for transport.

### Culture and sensitivity pattern of bacterial pathogens

The remainder of the swab was placed into Amies transport medium and transported on ice to the Clinical Laboratory at UPMC Children’s Hospital of Pittsburgh within 48 h and plated on blood and chocolate agars. Identification of SPN, HFLU, and MCAT on culture was accomplished using standard microbiological techniques. HFLU isolates were tested for the beta-lactamase production using a cefinase disk.

### qRT-PCR for viral co-infection

Using an aliquot of Amies transport media plus MagMax lysis/binding buffer, nucleic acid extraction was performed for viral identification using the ABI MagMax96 Express automated instrument and the MagMax 96 Viral Isolation Kit (Thermo Fisher, AMB 18365) [23, 24]. Adenovirus, influenza subtypes A/B/C, human metapneumovirus (MPV), human rhinovirus (HRV), parainfluenza virus (PIV) subtypes 1–4, Enterovirus D68, and respiratory syncytial virus (RSV) were tested for using individual real-time qRT-PCR assays. A Ct threshold of 40 was used for all viruses and positive and negative controls were included in each run.

### RNA-seq library generation, sequencing, and data processing

RNA was assessed for quality using a Fragment Analyzer 5300 and RNA concentration was quantified on a Qubit FLEX fluorometer. Libraries were generated with either the Illumina TruSeq Stranded Total RNA prep (20,020,599) or the Illumina Stranded Total Library Prep kit (Illumina: 20,040,529) according to the manufacturer’s instructions, after using the Illumina Ribo-Zero Plus rRNA Depletion Kit (20,037,135). Batch 5 was additionally treated with the Illumina Ribo-Zero Plus Microbiome rRNA Depletion Kit (20,072,062). For library generation, 100 ng of input was used for the Illumina TruSeq Stranded Total RNA protocol with 15 cycles of indexing PCR, and 20–100 ng of RNA input was used for the Illumina Stranded Total Library Prep protocol with 15 cycles of indexing PCR for 100 ng of RNA input and 17 cycles of indexing PCR for input RNA ≤ 100 ng. Library quantification and assessment was done using a Qubit FLEX fluorometer and the Fragment Analyzer 5300. Libraries were normalized and pooled to 2 nM by calculating the concentration based off the fragment size (base pairs) and the concentration (ng/μl) of the libraries. Sequencing was performed on an Illumina NextSeq 2000, using a P3 200 flow cell with sequencing read lengths of 2 × 101 bp, with a target of 40 million reads per sample. Sequencing of the nine control samples was done separately using an Illumina Novaseq 6000 with 2 × 101 bp read lengths. Sequencing data was demultiplexed by the Illumina on-board DRAGEN FASTQ Generation software. Library generation and sequencing was performed by the University of Pittsburgh Health Sciences Sequencing Core (HSSC), Rangos Research Center, UPMC Children’s Hospital of Pittsburgh, Pittsburgh, Pennsylvania, USA.

Fastp v0.23.1 [25] was used for quality trimming and adapter removal on default parameters. FastQC v0.11.9 [24] and MultiQC v1.12 [26] were used to check the quality of all sequence files before and after processing to ensure data was ready for analysis.

### Taxonomic classification of RNA-seq reads for detection of bacterial and viral pathogens

Taxonomic classification of sequencing reads was performed using Kraken 2 v2.1.2 [27] with default parameters. The PlusPF database dated 9/8/2022 (https://benlangmead.github.io/aws-indexes/k2) was used with Kraken 2, which was originally built from NCBI RefSeq archaeal, bacterial, viral, plasmid, human, UniVec_Core, protozoan, and fungal sequences. A Kraken 2 detection threshold of 3 reads was used for bacterial species (selected based on F1 score optimization), while no threshold was used for viruses. New pathogens identified by Kraken 2 but not included in the clinical panel were further validated using BLAST [28], MASH [29] and metAnnotate [30], focusing on samples associated with the largest estimated abundance for each pathogen.

The normalized abundance of each taxon was calculated as the number of reads per million (RPMs). Relative abundance heatmaps were generated using R v4.2.1 and the pheatmap v1.0.12 package. For display, log_10_(RPM + 1) values were used to avoid log(0) errors. Receiver operator curves (ROCs) were also generated in R and the area under the curve (AUC) was computed using the pROC package. Pathogen abundance jitter plots and top species plots were generated using ggplot2 v3.5.1 in R [31].

Viral load was estimated from RNA-seq data following the method of Graf et al. [32]. The number of detected reads for a virus was divided by the total number of reads in the sample and the size of the respective viral genome in kilobases, and then multiplied by 1 million to generate an RPKM value (reads per kilobase of reference sequence per million total sequencing reads).

### Microbiome analysis and quantification of bacterial gene expression

To explore beta-diversity across all samples, principal coordinates analysis (PCoA) was performed using Bray–Curtis dissimilarities computed from the Kraken2-predicted taxonomic profiles using the vegan v2.6–8 package in R. Alpha diversity was calculated for each sample using the Shannon index and compared across groups using the Kruskal–Wallis rank sum test. A pairwise comparison was also done between groups containing pathogens (viral plus bacterial) and no pathogens using the Wilcoxon rank sum test. Species enrichment analyses were performed by comparing abundances between the group of interest (e.g., all samples containing a bacterial pathogen) and the comparison group (samples with no pathogen detected). Fold-changes and *p*-values were calculated using R, with *p*-values calculated based on Wilcoxon rank sum tests.

Bacterial gene expression analysis was performed using Salmon v1.7.0 [33] by mapping RNA-seq reads to reference genomes of *H. influenzae* (NZ_CP085952.1), *S. pneumoniae* (NZ_LN831051.1), and *M. catarrhalis* (NZ_CP018059.1) obtained from NCBI Genbank. Transcript counts for all coding sequences were computed and imported into R using txImport v1.30.0 [34], and the mean expression levels in pathogen-positive samples (i.e., separately for MCAT + , SPN + , and HFLU +) were visualized as jitter plots using ggplot2 v3.5.1. Weakly expressed genes were identified as those with an average TPM < 10. GO term enrichment analysis was performed using a custom R script that compares GO term frequencies between the most highly expressed genes (upper quartile) and background frequencies, with GO annotations obtained from the original GFF3 files. Fisher tests were performed to calculate *p*-values*,* which were FDR-adjusted using the p.adjust function in R.

### Detecting beta-lactamase genes using RNA-seq

For the samples that were positive for HFLU based on culture tests, sequencing reads classified as non-human by Kraken 2 were extracted using extract_kraken_reads.py and assembled into contigs using the rnaSPAdes v3.15.4 with default parameters [35]. Using CARD resistance gene identifier (RGI) software v6.0.1 [36] and default database, the contigs were analyzed with the “main” function of the RGI tool with the “low-quality” and “include-nudge” parameters. The results were filtered to keep “strict” or “perfect” hits to beta-lactamase genes, genes acting on antibiotics belonging to the penam drug class, and hits with at least 10.0% sequence coverage to the reference gene.

### Viral genome reconstruction and phylogenetic analysis

RefSeq genomes for all viruses of interest were downloaded from NCBI. Non-human reads were mapped to viral genomes using BBMap v38.86 [37] to create.bam files. Mapping-based viral consensus sequences were reconstructed using samtools mpileup v1.16.1 with the ‘-a’ option. Zero-depth positions were kept but converted to Ns in consensus sequences. A python script was used to calculate whole genome coverage relative to the RefSeq viral genome. Genome coverage was considered complete if ≥ 99.5%. FastANI v1.32 was used to calculate the average nucleotide identity to the closest reference genome for each reconstructed genome.

The mapping-based viral consensus sequences from above were queried against the complete NCBI non-redundant nucleotide database using BLAST [28]. Up to 35 top matching sequences were downloaded and aligned to the reconstructed genome using the MUSCLE algorithm [38]. The multiple genome alignment was used to generate a phylogenetic tree with FastTree v2.1.10 [39], and FigTree v1.4.4 was used for tree visualization.

### Host response gene expression analysis

Host transcript abundance quantification was performed using Salmon v1.7.0 [33] with the Human Gencode v39 reference transcriptome, and the –validateMappings, –seqBias, and –gcBias flags. Differential gene expression analysis was performed using DESeq2 and tximport in R [40]. Related statistical analyses are described in the following section. Heatmaps were produced in R using pheatmap, v1.0.12 jitter plots using ggplot2 v3.5.1, and volcano plots using the EnhancedVolcano package v1.14.0.

### Cell type enrichment analysis

Cell type enrichment analysis was performed using xCell v1.1.0 [41] as implemented in the webserver at https://comphealth.ucsf.edu/app/xcell. Non-significant enrichment values (> 0.2) were omitted and only cell types with adjusted *p* values < 0.1 were explored. Heatmaps of cell type enrichment scores were generated using R v4.2.1 and pheatmap v1.0.12.

### Statistical analysis

Differentially expressed genes (DEGs) were detected by comparing samples positive for viruses only versus samples positive for bacteria only based on culture or qRT-PCR testing. In the design formula for the “DESeqDataSetFromTximport” function, we also controlled for potential confounding variables “batch number,” “sex,” and “age (scaled)”. Log2 fold changes and adjusted *p*-values (*q*-values) were calculated using the p.adjust function (Benjamini–Hochberg correction) in R for all genes, and a significance threshold of *q* ≤ 0.05 was used to identify DEGs. Function enrichment analysis of genes with significantly increased expression in the viral and bacterial groups was performed using EnrichR [42] (accessed June, 2023) with the GO Biological Process 2021 ontology and an FDR threshold of 0.05. For remaining batches 2–5, potential batch effects were examined using PCA and by quantitative comparison of viral and bacterial abundance values. No remaining batch effects were detected visually or quantitatively (Additional File 1: Fig S1).

A power analysis of the RNA-seq dataset was done using the RnaSeqSampleSize R package. A power analysis was conducted using the smallest group size in our cohort (*N* = 31 samples with bacterial infections), and the following parameters which were measured for our dataset (rho = 3, lambda0 = 24.88, phi0 = 12.4, f = 0.05, alpha = 0.01, m = 39,242).

### Construction of host response classifier and cross-validation

To perform feature selection, we started with the viral (*n* = 273) and bacterial (*n* = 548) upDEGs as candidate features. We then computed their variance-stabilized transformed (VST) expression levels across all samples, and compared these expression levels across the two groups using two-sample *t*-tests to derive *p*-values. The top 25 viral and top 25 bacterial genes based on *p*-value were then further filtered to remove correlated genes, pseudogenes, and non-protein-coding genes (e.g., lincRNAs). Correlated gene pairs (Pearson *r* > 0.75) were detected using a correlation matrix computed with the Hmisc package v5.2.2, and the gene with the largest mean absolute correlation was removed with the “findCorrelation” function. The above procedure resulted in a 10-gene set associated with bacterial infections and an 8-gene set associated with viral infections.

Using the above gene sets, random forest classifiers were then built using the caret package (v7.0.1). Four independent models were trained to classify clinical viral-positive and RNA-seq viral-positive patients using the 8-gene viral signature, as well as clinical bacterial-positive and RNA-seq bacterial-positive patients using the 10-gene bacterial signature. The default ntree parameter value of 500 was used and the recommended mtry parameter value of 3 was chosen as it is nearest to the square root of the number of features (*n* = 8 and *n* = 10). Ten-fold cross-validation was implemented via the `trainControl` function (`method = "cv", number = 10`). This procedure divided the dataset into 10 subsets, iteratively training on 9 subsets while testing on the remaining one. Probability predictions were enabled (`classProbs = TRUE`), and classification performance (sensitivity, specificity, and AUC) was assessed using the `twoClassSummary` function. The final AUC was calculated as the mean AUCs of the 10 cross-validation iterations.

## Results

### Cohort characteristics

**Fig. 1:**
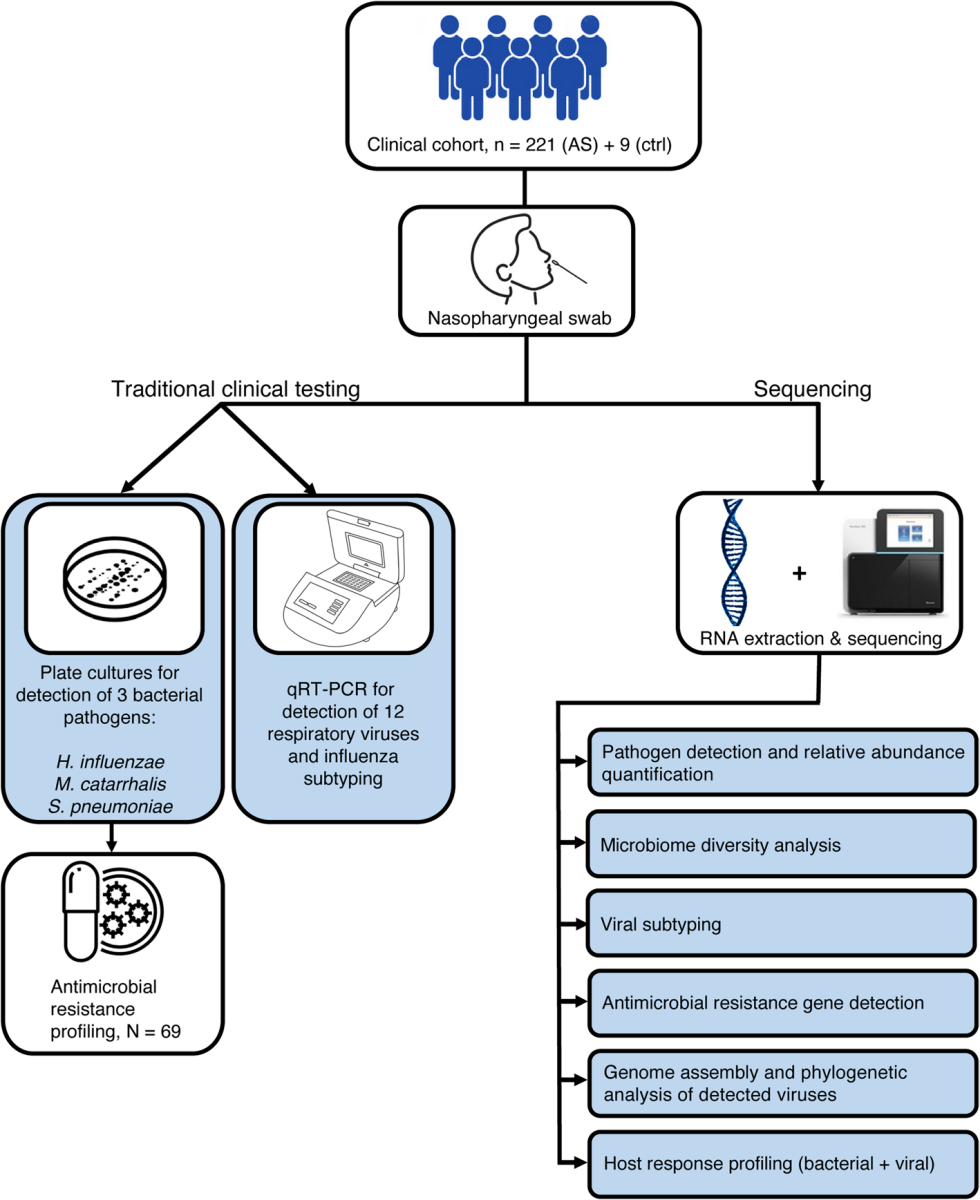
Overview of study design. The study cohort was comprised of 221 children with acute sinusitis who underwent collection of NP swabs. Culture was used to detect three bacterial species (*Haemophilus influenzae*, *Streptococcus pneumonia*, *Moraxella catarrhalis*) and qRT-PCR was used to detect 12 viruses of clinical relevance. *Haemophilus influenzae* isolates were tested for beta-lactamase production (*N* = 69). Parallel to this, RNA extraction from NP swabs and sequencing was also done to conduct metatranscriptomic analysis using a bioinformatics approach. Using the sequencing data, several analyses were performed: pathogen detection and quantification, reconstruction of detected respiratory viruses, detection of beta-lactamase genes, and transcriptomic analysis of host responses

A subset of 221 pediatric patients presenting with symptoms of acute sinusitis from a previous study [6] (Feb 2016 to Mar 2022), as well as nine healthy individuals as controls (Apr 2021 to June 2022), were selected for NP RNA-seq (Fig. 1, Table 1). Further details are provided in the Methods and in Shaikh et al. [6]. One naris was sampled using a NP swab and this was used for viral qRT-PCR, bacterial culture, and RNA sequencing [23]; 171 (77%) and 169 (76%) of the children tested positive for at least one bacteria or virus, respectively. Thirty-three children were positive for bacteria but not virus, and 31 were positive for virus but not bacteria. Parents assessed symptom severity daily during the 10 days following diagnosis. A power analysis of our RNA-seq dataset revealed an estimated 96% power to detect differentially expressed genes with fold changes ≥ 2 and assuming a group size of *N* = 31 (minimum used in later analyses) (see Methods).

**Table 1:** Demographic and clinical characteristics of pediatric patient participants with sinusitis. Demographic and clinical data for study cohort comprised of 221 children with persistent or worsening symptoms consistent with a diagnosis of acute sinusitis. Pathogen detection for 3 common bacteria and a panel of 14 viruses was accomplished using culture and qRT-PCR, respectively

Demographics	
Age (years)^a^	4.8 (3.3–6.4)
Gender	
Male	115
Female	106
**Clinical characteristics at time of diagnosis**	
Number of days with symptoms^a^	14 (9–16)
Fever at any time during the illness	121
History of asthma	39
History of allergic rhinitis	64
Coloured nasal discharge	148
**Clinical lab test results at time of diagnosis**	
One or more bacteria detected	171
One or more viruses detected	169
Positive for beta-lactamase^b^	27

^a^Median (interquartile range),

^b^Only samples positive for Hflu were tested (*N* = 69)

### Bacterial pathogen detection by metatranscriptomic analysis of NP samples

**Fig. 2:**
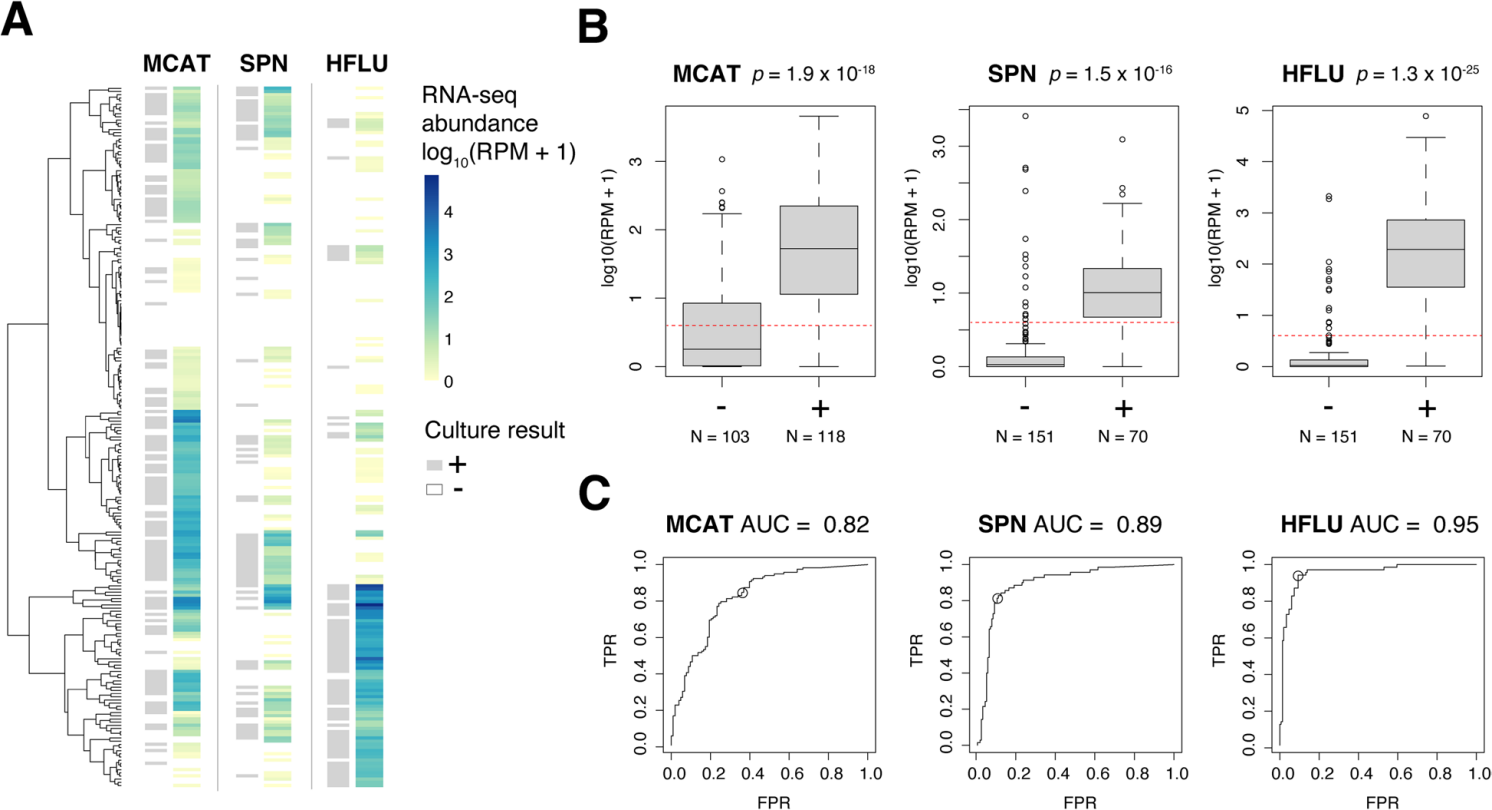
Metatranscriptomic detection of bacterial pathogens in NP samples from children with clinically diagnosed acute sinusitis.** A** Heatmap showing the detected abundance of three bacterial pathogens (*H. influenzae*, *M. catarrhalis*, *S. pneumoniae*) in patient metatranscriptomes. For each bacterium, the culture-based test result (positive—grey, negative—white) is shown on the left of the column, and the estimated RNA-seq abundance is depicted on the right of the column as a color gradient (absent—white, low—yellow, high—dark blue). Each row in the heatmap and tip in the hierarchical tree corresponds to an individual patient sample. **B** Boxplots depicting pathogen abundance in positive ( +) versus negative ( −) samples (labeled on *X* axis) defined based on culture. The boxes show the interquartile range and median line, and the whiskers show the variability extending to the furthest data points within 1.5 times above and below the interquartile range. Outliers outside of these ranges are shown as data points. Two-tailed *t*-test *p*-values for positive versus negative samples are shown above each plot. Red dashed lines indicate the detection threshold equivalent to 3 RPM. **C** ROC curves illustrating specificity and sensitivity of metatranscriptomic pathogen detection with AUC values displayed above. In each ROC curve, the circled data point indicates the true positive rate (TPR) and false positive rate (FPR) associated with the 3 RPM threshold

To identify potential bacterial and viral pathogens in the 221 samples, we performed metatranscriptomic sequencing of RNA derived from NP swabs. First, we aimed to quantify the abundance of three bacterial pathogens of interest—*S. pneumoniae* (SPN), *M. catarrhalis* (MCAT), and *H. influenzae* (HFLU)—as these pathogens are commonly isolated in children with bacterial sinusitis^4^. We note that our use of the term “pathogen” does not imply that these organisms are necessarily the causative agents of sinusitis infections. After quality filtering, we performed taxonomic classification of the sequencing reads using Kraken 2 [27]. The relative abundance of the three bacterial pathogens (shown in Fig. 2A) was calculated based on the normalized abundance of reads (reads per million, RPM) that mapped to each species. One or more of these three bacterial pathogens were detected in a total of 177 patients (80%). Two or more bacterial pathogens were detected in 89 (40%) patients, and 25 (11%) of patients had all three bacterial pathogens detected. On an individual basis, SPN was detected in 73 (33%), MCAT in 137 (62%), and HFLU in 81 (37%) of patient samples. The clinical culture and RNA-seq-based results for bacterial detection for each patient are included in Additional File 2: Tables S1 and S2.

Next, we examined the extent that the calculated abundance of these bacterial pathogens from RNA-seq agreed with their presence/absence based on culture. For all three pathogens, we detected a significant increase in RNA-seq abundance in those with a positive culture (all *p* values < 1 × 10^−15^), demonstrating concordance between the metatranscriptomic data and culture (Fig. 2B). Some pathogen-negative samples based on culture had an RNA-seq pathogen abundance greater or equal to the mean abundance seen in positive samples. We then assessed the ability of the RNA-seq data to predict the culture-based test results for each pathogen, and generated ROCs by varying the detection threshold (Fig. 2C). HFLU infections could be detected with the highest accuracy by RNA-seq with an AUC of 0.95, SPN infections with an AUC of 0.89, and MCAT infections with an AUC of 0.82. Using a threshold of 3 reads per million, HFLU was detected with a sens/spec of 94%/90%, SPN with 81%/89% and MCAT with 85%/64% (Table 2). Additionally, none of the nine negative control patients had detectable HFLU or SPN at these thresholds, while 2 patients (22%) had detectable MCAT (Additional File 1: Fig S2A).

**Table 2:** Sensitivity and specificity metatranscriptomics for detection of bacteria identified by culture or viruses identified by qRT-PCR

	**Sensitivity (%)**	**Specificity (%)**
**Bacteria**		
* Moraxella catarrhalis* (MCAT)	85	64
* Streptococcus pneumoniae* (SPN)	81	89
* Haemophilus influenzae* (HFLU)	94	90
**Viruses**		
Influenza A (INFA)	100	94
Influenza B (INFB)	100	97
Influenza C (INFC)	33	96
Human metapneumovirus (MPV)	100	91
Respiratory syncytial virus (RSV)	90	92
Human rhinovirus (HRV)	73	77
Parainfluenza virus 1, Human respirovirus 1 (PIV1)	100	94
Parainfluenza virus 2, Human orthorubulavirus 2 (PIV2)	100	99
Parainfluenza virus 3, Human respirovirus 3 (PIV3)	100	91
Parainfluenza virus 4, Human orthorubulavirus 4 (PIV4)	91	91
Adenovirus (ADV)	44	97
Enterovirus D68 (EVD68)	100	90

### Beta-lactamase gene detection in HFLU-positive samples

We next examined whether metatranscriptomics could identify potential resistance genes associated with HFLU. Culture-based tests for beta-lactamase were performed for all HFLU-positive samples, and these were used as the reference standard to analyze the accuracy of RNA-seq-based detection. We assembled all non-human reads from samples that were clinically positive for HFLU (*N* = 69) and used the Comprehensive Antibiotic Resistance Database (CARD) [36] to detect beta-lactamase genes with at least 10% coverage (Additional File 1: Fig S3). Beta-lactamase genes were detected in 74% (20/27) of the samples associated with resistant HFLU, and in 33% (13/42) of the samples associated with non-resistance HFLU, which reflects a significant (2.1-fold) increase in detected beta-lactamase genes in the resistant samples (*p* = 0.002, Fisher exact test). The complete list of genes and the portion of the reference genome detected for each hit can be found in Additional File 2: Tables S3-S5.

### Metatranscriptomic detection and analysis of respiratory viruses

To examine the ability of metatranscriptomics to detect viral infections, we first focused on respiratory viruses identified using qRT-PCR. Viruses tested for included influenza A (INFA), influenza B (INFB), influenza C (INFC), human metapneumovirus (MPV), human rhinovirus (HRV, which tested for rhinovirus types A, B, and C), parainfluenza virus 1 (PIV1), parainfluenza virus 2 (PIV2), parainfluenza virus 3 (PIV3), parainfluenza virus 4 (PIV4), respiratory syncytial virus (RSV, types A and B), human adenovirus (ADV), and enterovirus D68 (EVD68). One or more viruses were detected by metatranscriptomics in 175 patients (79%), two or more in 101 patients (46%), and three or more in 36 patients (16%). HRV was detected most frequently (45%), followed by MPV (14%) and INFA (13%).

**Fig. 3:**
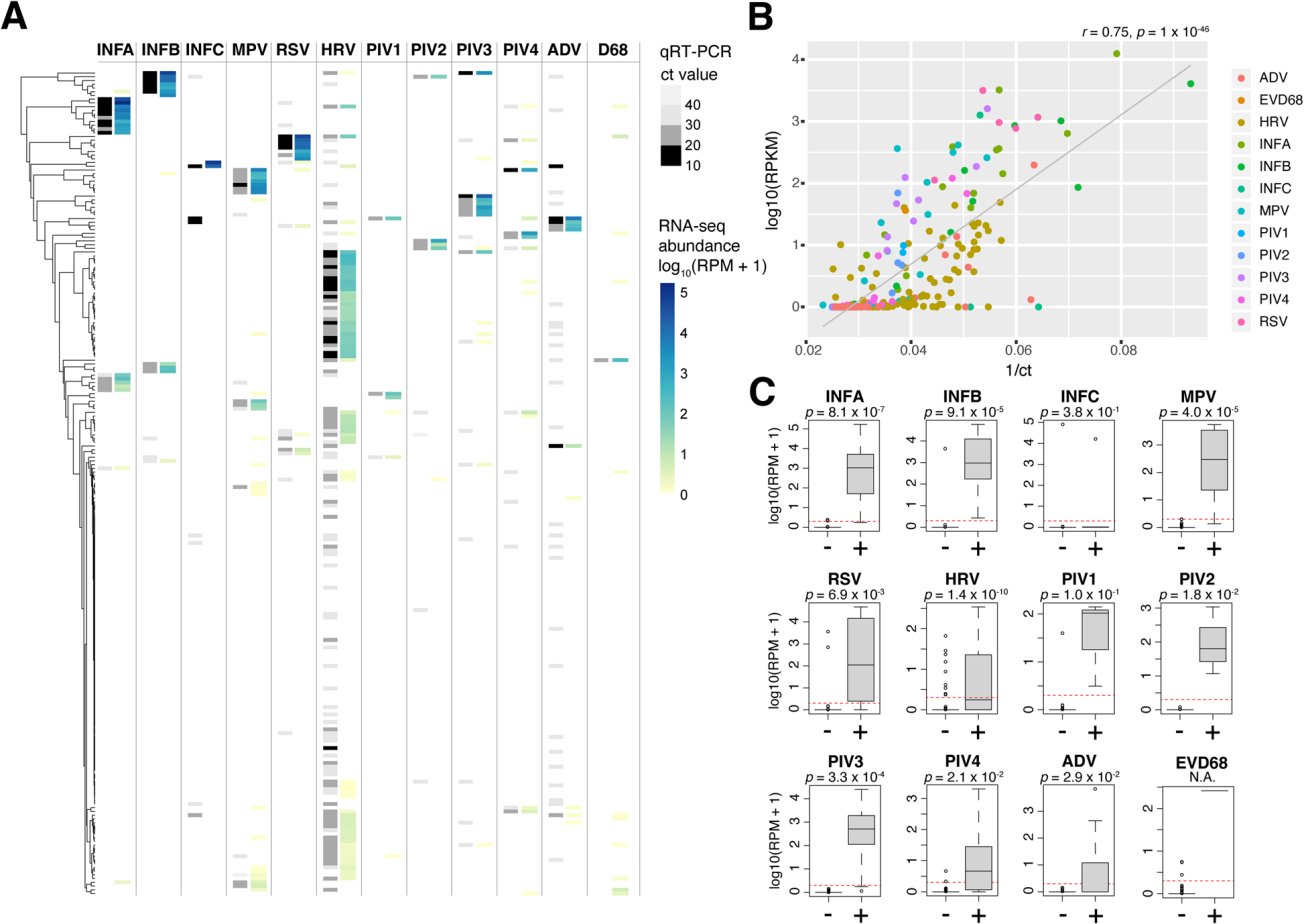
Detection of common respiratory viruses in NP metatranscriptomes.** A** Abundance heatmap for viruses detected in NP metatranscriptomes for 221 patients. For each virus, the qRT-PCR result is shown on the left of the column as a color gradient (negative—white, high to low cycle threshold values—light gray to black), and the estimated RNA-seq abundance is depicted on the right of the column as a color gradient (absent—white, low—yellow, high—dark blue). Each row in the heatmap and tip in the hierarchical tree corresponds to an individual patient sample. **B** qRT-PCR abundance (1/ cycle threshold) versus metatranscriptomic viral load (log_10_ of the RPKM). The estimated viral load from RNA-seq is significantly correlated with 1/Ct value from qRT-PCR. **C** Metatranscriptomic abundance of respiratory viruses in negative ( −) versus positive ( +) samples (labelled on *X* axis) defined by qRT-PCR test result. The boxes show the interquartile range and median line, and the whiskers show the variability extending to the furthest data points within 1.5 times above and below the interquartile range. Outliers outside of these ranges are shown as data points. Two-tailed *t*-test *p*-values for positive versus negative samples are shown above each plot. Red dashed lines indicate the detection threshold equivalent to 1 RPM

Next, we examined the extent that the RNA-seq-based predictions matched viral presence/absence based on the qRT-PCR. As shown visually in Fig. 3A, the relative abundance of viruses detected by metatranscriptomics was in strong agreement with the results of qRT-PCR-based tests, with lower qRT-PCR cycle threshold (Ct) values corresponding to higher RPM values in RNA-seq. A significant correlation (*r* = 0.75, *p* = 1.3 × 10^−46^) was detected between 1/Ct values and viral load calculated as log_10_(reads per kilobase million, rpkm) [32] (Fig. 3B), and this relationship was significant (*p* < 1 × 10^−5^) for all viruses (Additional File 2: Table S6). However, some viruses (e.g., ADV, HRV) had weaker correlations (*r* < 0.5). Samples containing viruses detected by qRT-PCR but not by RNA-seq had significantly higher cycle thresholds (mean = 34.7) compared to true positives (mean = 23.2; *t*-test *p*-value = 5.5 × 10^−5^), which has been reported in previous RNA-seq studies [43]. For nine viruses, we detected a significant (*p* < 0.05) increase in metatranscriptomic abundance in those with a positive qRT-PCR result (Fig. 3C). The three non-significant cases (INFC, PIV1, EVD3) were viruses detected in very few (1 to 6) individuals, limiting statistical power.

We then calculated the accuracy of viral detection by using the results of the qRT-PCR tests as the ground truth. Due to the uniqueness of viral sequences, we found that a very low threshold (≥ 1 RPM) was sufficient to distinguish virus-positive from negative samples. Using this threshold, we calculated the sensitivity and specificity of metatranscriptomic pathogen detection for each of the 12 viruses as shown in Table 2. Nine out of the 12 viruses were detected with 90–100% sensitivity and specificity, while INFC, HRV, and ADV were detected with lower accuracy. Additionally, none of the 12 viruses were detected in the negative control samples. Overall, we were able to detect the 12 viruses with an average sensitivity/specificity of 86%/92%. These accuracies are consistent with other studies performing sequencing-based pathogen detection using NP samples [32, 43].

### RNA-seq uncovers additional pathogens and alternate explanations of disease etiology

**Fig. 4:**
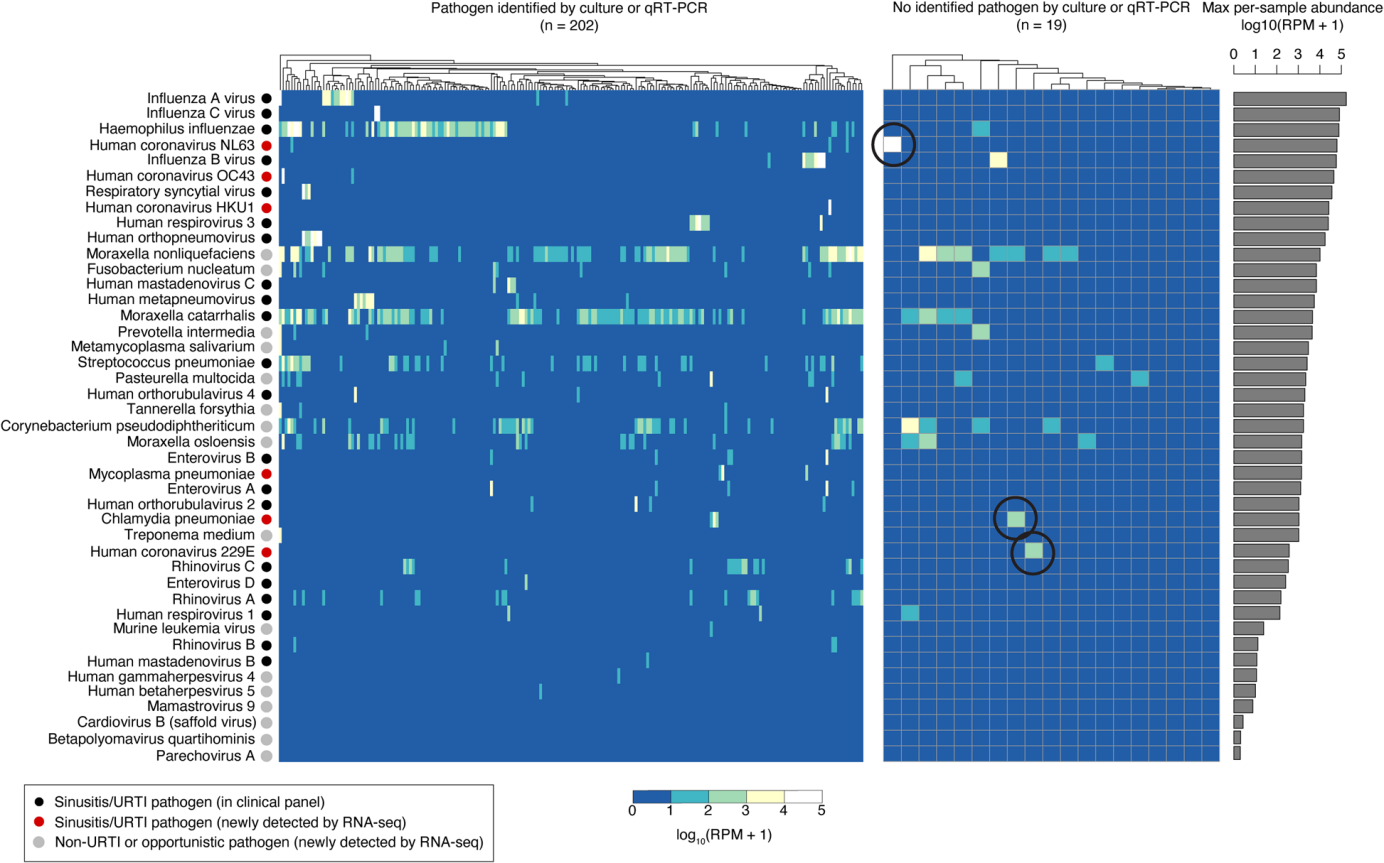
Metatranscriptomics of NP samples from children with acute sinusitis identified organisms not detected byqRT-PCRor culture. The organisms included in the heatmap are a subset of the full set of organisms detected by RNA-seq that exceed minimum abundance thresholds and include human pathogenic bacteria and viruses (see Additional File: Table S7 for full dataset). The organisms are sorted vertically based on their maximum relative abundance within a sample (across 221 samples). The heatmap displays the relative abundance of each organism in each sample as estimated by Kraken 2. The left heatmap includes samples with clinically identified pathogens by qRT-PCR or culture (*N* = 202), and the right heatmap includes 19 samples without a pathogen detected by qRT-PCR or culture. For the latter samples, several samples contain additional organisms identified by metatranscriptomics that are plausible causes of sinusitis. The barplot on the right depicts the maximum relative abundance of each pathogen across all samples

By sequencing total RNA within a sample, metatranscriptomics has the potential to detect additional pathogens beyond those tested by culture or qRT-PCR. We therefore screened our RNA-seq dataset for additional pathogens previously associated with URTIs and/or sinusitis infections, as well as non-URTI pathogens and opportunistic pathogens, and further validated the identified species using additional bioinformatic approaches (see Methods). Across the 221 patient samples, we detected 22 additional pathogens that were not tested for clinically, including 11 bacteria and 11 viruses (Fig. 4, see Additional File 2: Table S7 for abundance profiles). These species were then ranked in terms of their maximum relative abundance within a sample (Fig. 4).

Newly identified bacterial pathogens include fourteen species listed in Fig. 4. The most notable identifications include *Mycoplasma pneumoniae* and *Chlamydia pneumoniae*, which were not included in the clinical panel but have been previously implicated in pediatric sinusitis and URTIs [44, 45]. In addition, opportunistic pathogens including *Fusobacterium nucleatum*, *Moraxella* spp., and others were also detected (Fig. 4), but some of these likely have a commensal role in the nasopharynx. Interestingly, we also detected periodontitis-associated bacteria, *Treponema medium*, *Prevotella intermedia*, and *Tannerella forsythia* [46], in a few (*N* = 1 to 4) samples, and all three co-occurring in the same patient. Follow-up investigation of this patient revealed that they were admitted to an emergency room with a severe tooth infection 1 year after the NP swab sample was taken.

Newly identified viral pathogens with the highest abundance include four human coronaviruses known to cause upper respiratory infections (NL63, OC43, HKU1, and 229E)*.* We also detected parechovirus A and cardiovirus B (saffold virus), which have been associated with respiratory illness in children [47, 48], as well as other viruses that are not typically associated with respiratory infections including mamastrovirus 9, enteroviruses A and B, human gammaherpes virus 5, human betaherpes virus 5, and sequences related to murine leukemia virus (Fig. 4).

Of the 19 samples that had no pathogen detected by culture or qRT-PCR, 11 contained identified pathogens based on RNA-seq profiling. Three of the 11 samples (circled in Fig. 4) contained known pathogens detected at high abundance (ranging from ~ 250 to 60,000 RPM) that were not included in the clinical pathogen panel: the coronaviruses NL63 and 229E, and the bacterium, *Chlamydia pneumoniae*. Eight of the 11 samples had pathogens detected by RNA-seq at variable levels exceeding 10 RPM but not by qRT-PCR or culture, including influenza B (*N* = 1), parainfluenza virus 1 (*N* = 1), SPN (*N* = 1), MCAT (*N* = 4), and HFLU (*N* = 1).

Ultimately, these additional detected pathogens highlight the ability of RNA-seq to provide a more complete picture of the microbiome and virome present in acute sinusitis samples and suggest an expanded panel of viruses and bacterial pathogens to be used in future clinical workflows.

### Viral genome reconstruction and subtyping from host-derived metatranscriptomes

**Fig. 5:**
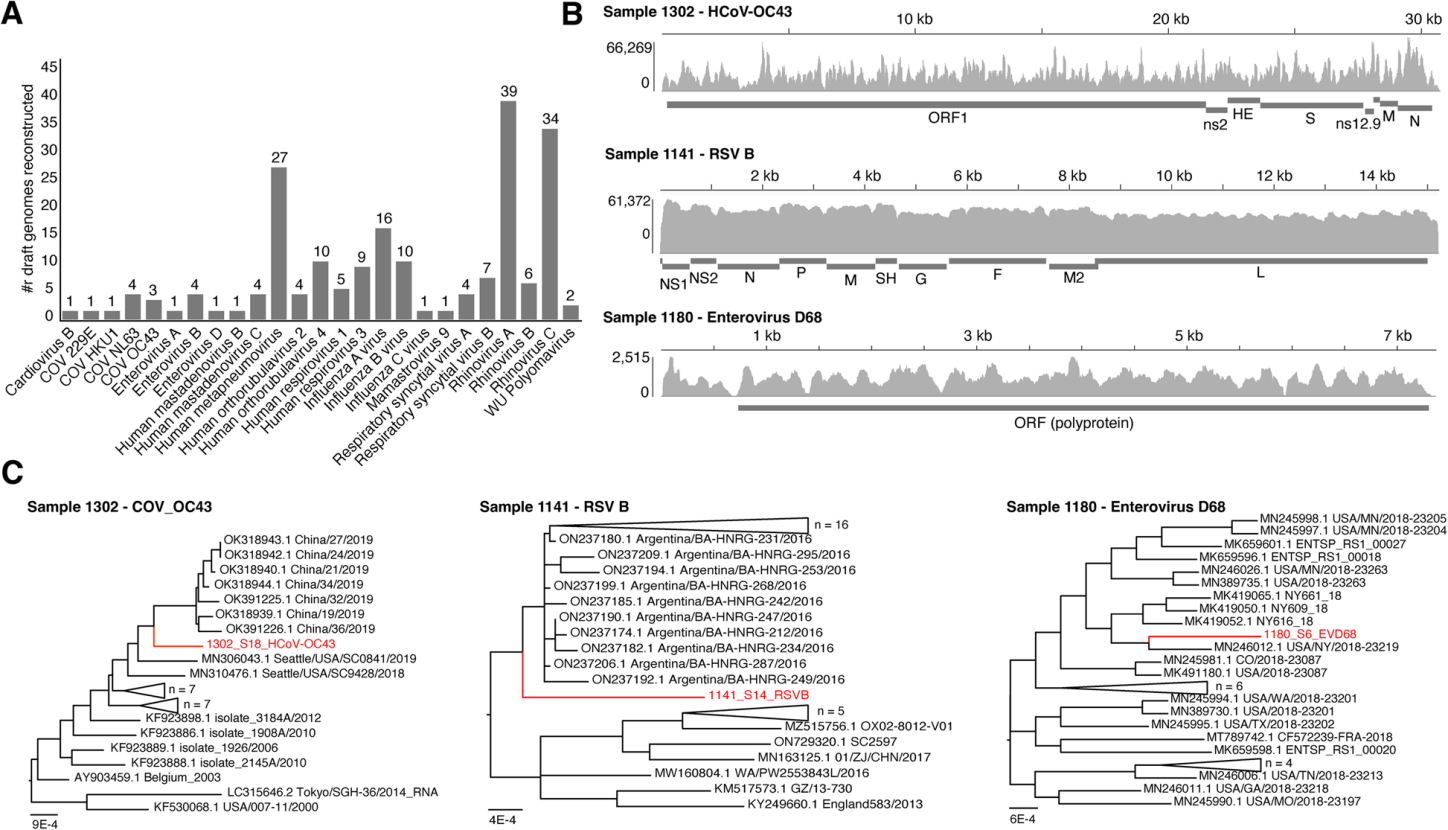
Reconstructed viralgenomes from children with clinically diagnosed acute sinusitis.** A** Bar graph depicting the number of reconstructed genomes for various species of respiratory viruses across the full dataset (*N* = 196 total viruses reconstructed from 163 samples). **B** Read pileups for three selected samples showing sequencing reads mapped to reference genomes of human coronavirus (HCoV) OC43 (NC_006213), RSV (NC_001781), and enterovirus D68 (NC_038308). **C** Phylogenetic analysis of three reconstructed viral genomes and their top 25 closest matching complete genomes from BLAST. Each newly reconstructed virus (red) is a unique strain that clusters as a distinct branch within its phylogenetic tree

By aligning the RNA-seq reads to reference genomes of identified viruses, we were able to reconstruct partial to complete genomes for a total of 196 viruses across 163 samples, including 25 different human pathogenic viruses (Fig. 5A). In addition to the 12 viral groups from the clinical panel (Fig. 3), genomes were reconstructed for 9 additional respiratory viruses (e.g., coronaviruses) not tested for clinically. We also reconstructed genomes of enterovirus A and B, WU polyomavirus which has been associated with respiratory infections [49], and mamastrovirus 9 which was identified in a gastroenteritis outbreak [50]. A total of 31 (15%) were 100% complete, while 60 (30%) had completeness > 90% (Additional File 2: Table S8). All reconstructed viral genomes were phylogenetically verified by sequence comparison to related genomes in NCBI through BLAST, with average nucleotide identities (ANIs) ranging from 95–100%.

To explore the use of reconstructed genomes for viral subtyping, we focused on the predictions for influenza A and B, since these were subtyped clinically using qRT-PCR. The subtyping results using reconstructed influenza genomes showed excellent agreement with the clinical results, with Influenza A subtypes H1N1 and H3N2 having 100% (15/15) agreement and Influenza B subtypes Yamagata and Victoria having 82% agreement (9/11) with qRT-PCR results (Additional File 2: Table S9).

We then focused on several cases of interest, performing a deeper genomic and phylogenetic analysis of newly reconstructed genomes. Three examples of reconstructed viral genomes are shown in Fig. 5B, including a genome of a novel HCoV-OC43 strain, an RSVB genome, and an enterovirus D68 genome. All three of these genomes are unique from other strains in the NCBI database (Fig. 5B) as they formed distinct lineages in phylogenetic analysis (Fig. 5C). All three of the genomes also showed broad sequencing coverage across the genome.

### Microbiome analysis of patient metatranscriptomes

Next, we focused on analysis of microbial diversity and gene expression patterns across all 221 samples. Although the majority reads were of human origin (99% average across datasets), the remaining 1% could be targeted by microbiome analysis (Additional File 2: Table S10). Microbial reads classified into bacterial (63.8%), viral (35.3%), and fungal species (2.5%), with viral abundance likely skewed by the high frequency of viral infections (*N* = 31, 14%). An abundance heatmap of the most abundant bacterial and viral species is included in Additional File 1: Fig S4. PCoA ordination plots revealed no clear pattern of clustering based on overall microbiome profiles (Additional File 1: Fig S5A). In addition, patients with viruses, bacterial pathogens, or both detected displayed similar levels of alpha diversity (Shannon Index) (Additional File 1: Fig S5B). However, patients with no pathogens detected had higher Shannon diversity levels than patients with pathogens (viral and/or bacterial) detected (*p* < 0.05).

A species enrichment analysis was performed to identify additional species that are associated with the bacterial or viral infections. This analysis re-discovered the expected species for bacterial infections (e.g., HFLU, SPN, and MCAT) and common viruses in viral infections (Influenza A and Rhinovirus A), but did not detect additional significant abundance shifts in the microbiome with *p* < 0.01 (Additional File 1: Fig S5C).

**Fig. 6:**
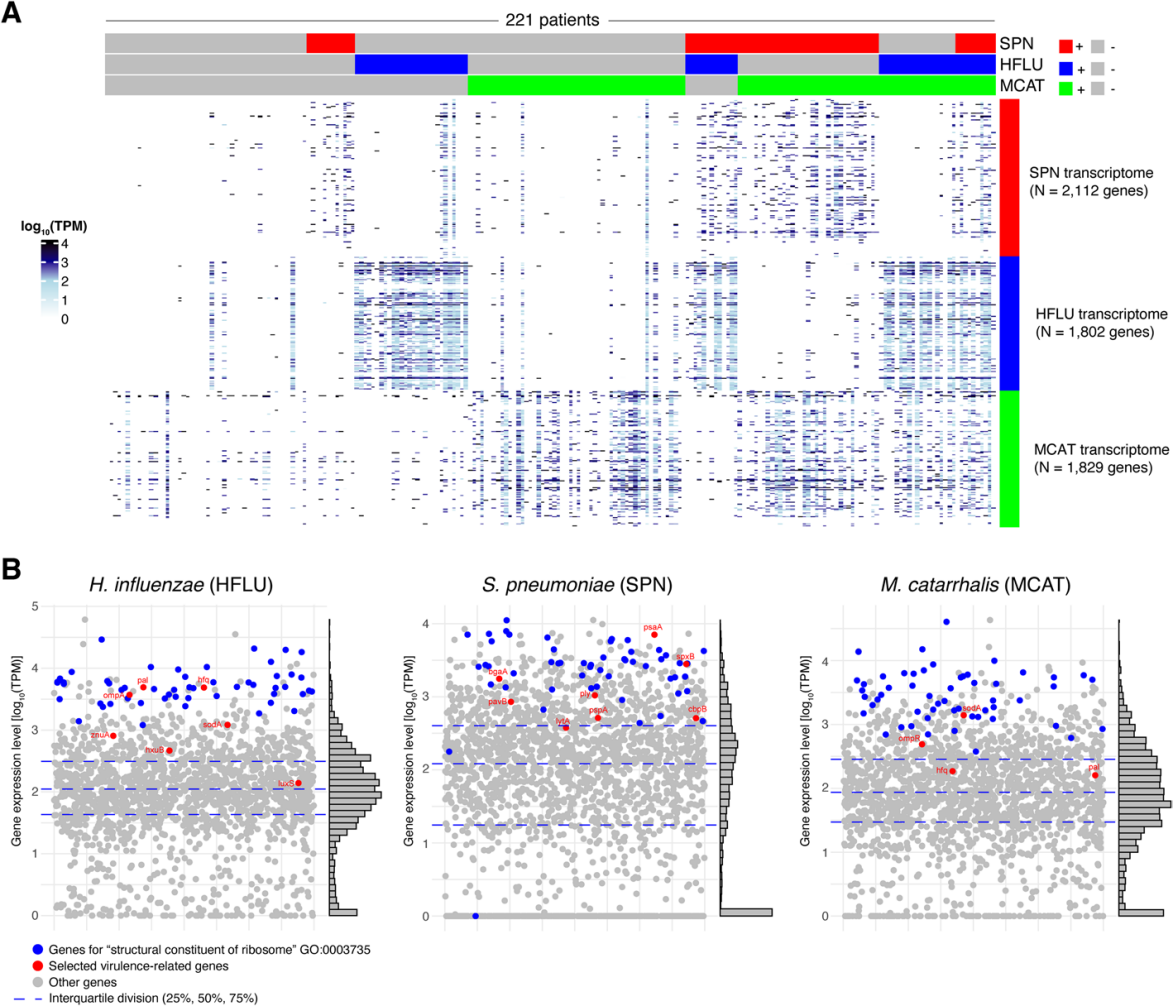
Transcriptomic analysis of bacterial gene expression in patients with SPN, HFLU, or MCAT detected by culture. **A** Gene expression levels of all SPN, HFLU, and MCAT genes (*Y* axis) across patients (*X* axis). Above the heatmap, patient samples have been ordered based on presence/absence of SPN, HFLU, and MCAT as detected by culture. The heatmaps reveal broad expression across the SPN, HFLU, and MCAT transcriptomes with high relative expression associated with bacteria-positive patients as expected. **B** Scatterplots of mean expression levels for individual HFLU, SPN, and MCAT genes across all HFLU, SPN, and MCAT positive patients, respectively. Genes with high relative expression (i.e., above the 75.th percentile) were investigated further and associated primarily with translation (ribosomal genes, colored blue) as well as genes encoding virulence factors (colored red)

We then analyzed the HFLU, SPN, and MCAT transcriptomes across all 221 patients to examine bacterial transcriptome coverage and patterns of gene expression (Fig. 6). As visualized in the heatmap shown in Fig. 6A, the bacterial transcriptomic expression profiles corresponded strongly with the culture results as expected (e.g., HFLU gene expression detected in HFLU positive patients). In addition, the analysis revealed broad transcriptomic coverage for each bacterial pathogen, with the majority of genes having detectable expression. In HFLU, SPN, and MCAT positive patients, 1606 (89% of HFLU genes), 1654 (78% of SPN genes), and 1599 (87% of MCAT genes) were detected by RNA-seq above minimum expression thresholds (Additional File 2: Tables S11-S13). For all three pathogens, GO-term enrichment analysis revealed that ribosomal genes were significantly enriched (*q* < 0.001) among highly expressed (upper quartile) genes (Additional File 2: Table S14). In addition to ribosomal genes, we also identified other key genes including virulence factors with high expression levels in the three pathogens. Virulence-related genes expressed at particularly high levels included *H. influenzae* genes *ompA* (outer membrane protein A) and *hfq* (host factor I), *S. pneumoniae psaA* (pneumococcal surface adhesin A), *spxB* (pyruvate oxidase), *bgaA* (beta-galactosidase), and *ply* (pneumolysin), and *M. catarrhalis* genes *sodA* (superoxide dismutase) and the response regulator, *ompR* (Fig. 6B). These results suggest that virulence genes relevant to bacterial infection as well as ribosomal genes are highly expressed by these bacterial pathogens during infection of the nasopharynx.

### Host-response expression profiles distinguish bacterial from viral infections

Although RNA-seq analysis was capable of detecting pathogens directly from reads, most reads within RNA-seq samples were host (human) derived, ranging from 64.7–99.9% (Additional File 2: Table S10), which enables host-response profiling to potentially identify host biomarkers and immune responses associated with disease etiology [15, 51–53].

Cell type enrichment analysis using xCell [41] revealed enrichments of specific immune cell types, including neutrophils, monocytes, and macrophages, across the samples (Additional File 1: Fig S6A). Although immune cell type enrichments showed significant variation across samples, other cell types such as epithelial cells showed a more uniform profile across all samples (Additional File 1: Fig S6A). We then analyzed the association between the enrichments of each cell type and presence/absence of bacterial and viral pathogens based on clinical testing. M1 macrophage enrichments had the highest accuracy (AUC = 0.75) for predicting patients with viruses detected, while neutrophil enrichments had the highest accuracy (AUC = 0.76) for predicting patients with bacterial pathogens (Additional File 1: Fig S6B).

**Fig. 7:**
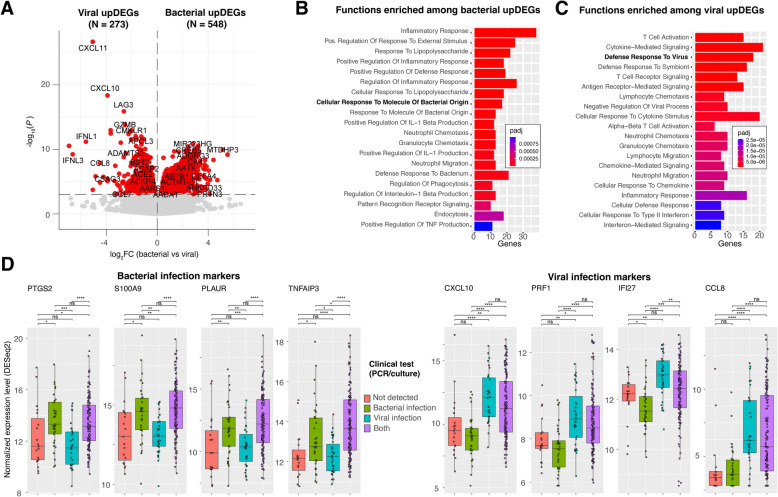
Identification of differentially expressed host genes indicative of host-responses to bacterial and viral infection in acute sinusitis patients.** A** Volcano plot of differentially expressed genes between samples with only bacterial pathogens and samples with only viral pathogens according to qRT-PCR and culture test results. Human genes shown in the upper right quadrant have significantly increased transcript abundance in samples with bacteria (bacterial upDEGs), and genes in the upper left quadrant have significantly increased transcript abundance in samples with virus(es). Genes are partitioned in the plot based on *p*-value significance thresholds. **B, C** Biological functions and pathways that are significantly enriched among bacterial and viral upDEGs, calculated using enrichR. For each function term, the associated adjusted *p*-value and number of genes is depicted. **D** Example bacterial and viral upDEGs and their expression levels (transcript abundance) across four categories of patients: those with neither bacteria nor virus detected by culture or qRT-PCR; those with only bacteria, those with only virus, and those with both a bacteria and virus. The results of Wilcoxon rank sum tests are shown for all pairwise comparisons (ns: *p* > 0.05; *: *p* ≤ 0.05, **: *p* ≤ 0.01; ***: *p* ≤ 0.001; ****: *p* ≤ 0.0001)

To further identify differentially expressed genes (DEGs) associated with bacterial versus viral infections, we compared host gene expression profiles of patients with bacterial pathogens to those with viral pathogens based on clinical diagnostic testing (culture/qRT-PCR) (Fig. 7A). Due to the presence of many (*N* = 138) complex samples containing a mixture of viral and bacterial pathogens, we chose to simplify the initial comparison and compared samples with only bacterial pathogens (*N* = 33) to those with only viral pathogens (*N* = 31) but subsequently analyzed all 221 samples. A total of 821 significant DEGs were detected with *q* < 0.001, of which 548 genes had increased expression in bacterial-positive patients and 273 genes had increased expression in viral-positive patients (Fig. 7A, Additional File 2: Table S15). We termed these genes as “bacterial upDEGs” and “viral upDEGs.”

Based on function enrichment analysis, bacterial upDEGs were significantly associated with neutrophil regulation, regulation of inflammatory response, response to lipopolysaccharide, and response to molecule of bacterial origin (Fig. 7B). Bacterial upDEGs included *PTGS2* (sixfold increase in bacterial-positive patients, *q* = 3.1 × 10^−7^), *S100A9* (fourfold increase, *q* = 4.2 × 10^−6^, *PLAUR* (fivefold increase, *q* = 7.3 × 10^−6^), *TNFAIP3* (fourfold increase, *q* = 1.3 × 10^−5^), *IL1A* (sixfold increase, *q* = 1.0 × 10^−4^), *IL1B* (sixfold increase, *q* = 4.0 × 10^−5^), *CXCL2* (fourfold increase, *q* = 1.3 × 10^−5^), and *NFKBIA* (fourfold increase, *q* = 1.8 × 10^−5^) (Fig. 7D).

Viral upDEGs were found to be significantly associated with cytokine signaling, defense response to virus, T cell receptor signaling, and inflammatory response (Fig. 7C), which are related to viral immune response pathways. Viral upDEGs included *CXCL11* which was increased 33-fold in virus-positive patients (*q* = 4.9 × 10^−23^), *CXCL10* (15-fold increase, *q* = 2.6 × 10^−15^), *CCL8* (23-fold increase, *q* = 2.3 × 10^−6^), *PRF1* (fourfold increase, *q* = 3.8 × 10^−9^), and *IFI27* (twofold increase, *q* = 8.5 × 10^−7^), which represent putative biomarkers of viral infection in our analysis (Fig. 7D).

In general, representative viral and bacterial upDEGs had lower expression levels for samples in which no bacteria or virus was detected by qRT-PCR/culture, and higher expression levels for samples containing both a virus and bacterial pathogen (Fig. 7D). Interestingly, there are several exceptions to this pattern including four samples that had a strong antiviral response despite there being no virus detected by qRT-PCR/culture. Deeper investigation of these samples by RNA-seq revealed that three of them contained respiratory viruses (two coronaviruses and influenza B) (Fig. 4B) that were not detected by the qRT-PCR tests. Other exceptions include two samples which had no bacterial pathogen detected by culture/qRT-PCR but had a strong antibacterial response. One of these samples (sample 1303) had a bacterial pathogen (MCAT) identified in high abundance by RNA-seq. These results suggest that host-response profiling may provide an indication of viral or bacterial infection when traditional tests fail to detect a pathogen.

### Magnitude of host responses correlates with viral and bacterial pathogen abundance

If the identified viral and bacterial upDEGs are genuine biomarkers of viral and bacterial infections, respectively, then their levels of expression should correlate with the abundance of viral and bacterial pathogens estimated from RNA-seq. To test this hypothesis, we calculated the total bacterial pathogen abundance as the sum of the relative abundance of the pathogens SPN, HFLU, and MCAT. We then binned all samples into ten groups, with group 1 having the lowest bacterial pathogen abundance, and group 10 having the highest. We then repeated this analysis for viral pathogens, summing the total abundance of 12 viral pathogens as well as the coronaviruses that were clearly present based on RNA-seq data, but missing from the clinical test.

**Fig. 8:**
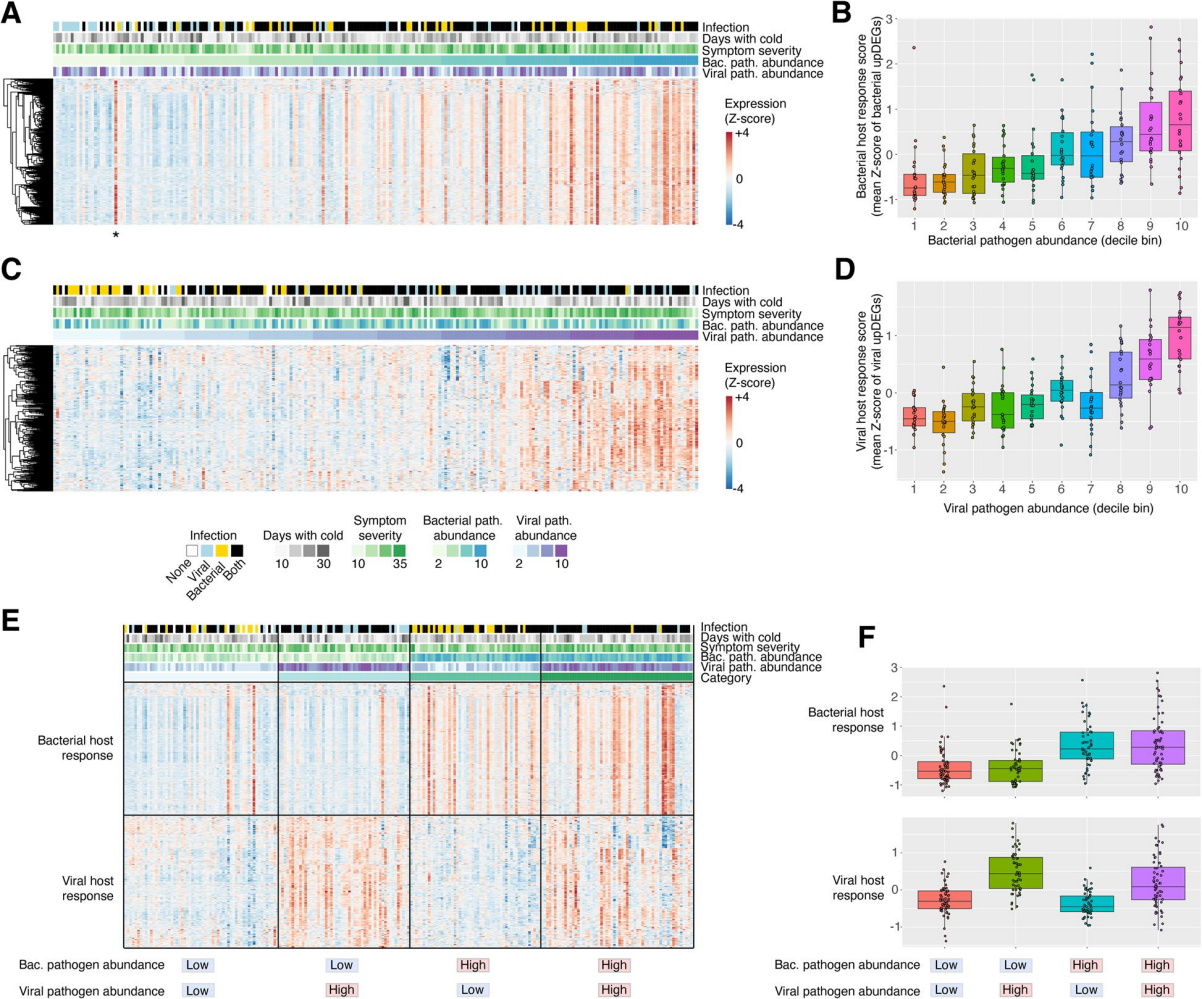
Host-response correlates with relative abundance of bacterial and viral pathogens.** A** Expression heatmap of bacterial upDEGs (bacterial host response genes), with samples (columns) sorted by total metatranscriptomic bacterial pathogen abundance. The associated metadata for all samples is also plotted above the heatmap. * Also shown is an outlier sample associated with a strong bacterial host response but with low detected abundance of MCAT, HFLU, or SPN. **B** Bacterial host response score versus metatranscriptomic bacterial pathogen abundance. The bacterial host response score was calculated as the mean expression level (*Z*-scores) of all the bacterial upDEG genes. **C** Expression heatmap of viral upDEGs (viral host response genes), with samples (columns) sorted by metatranscriptomic viral pathogen abundance. **D** Viral host response score versus metatranscriptomic viral pathogen abundance. The viral host response score was calculated as the mean expression level (*Z*-scores) of all the viral upDEG genes. **E** Heatmap of bacterial and viral host responses (upDEGs), where samples (columns) have been sorted into four groups based on high or low bacterial/viral pathogen abundance, with high considered as a 60th percentile or greater relative abundance. In general, samples with low bacterial and viral abundance tend to lack a bacterial/viral host response, whereas samples containing bacteria, viruses, or both displayed the appropriate response. **F** Jitter plots of the bacterial and viral host response scores across four categories of samples. Bacterial and viral host response scores were calculated by averaging the expression level *Z*-scores of all bacterial and viral upDEGs, respectively

As shown in Fig. 8A, with increasing abundance of bacterial sinusitis pathogens (MCAT, SPN, HFLU), there is a clear increase in expression levels of bacterial upDEGs. To quantify this pattern, for each sample we calculated the “magnitude” of the bacterial and viral host response as the average expression level (*Z*-score) of the bacterial and viral upDEGs. As shown in Fig. 8B, the magnitude of bacterial host response correlated significantly with bacterial pathogen abundance (Pearson *r* = 0.50, two-tailed *p* = 1.6 × 10^−15^). The same pattern was also seen for viruses: that is, the abundance of viral pathogens also correlated significantly with the magnitude of viral host-response (Pearson *r* = 0.33, two-tailed *p* = 5.8 × 10^−7^) (Fig. 8C,D). This trend is also apparent by the distributions of bacterial and viral host-response scores for each clinical group (culture/qRT-PCR testing) including the nine negative controls, which showed the same pattern as the samples with no pathogens detected (Additional File 1: Fig S2B). Both the bacterial and viral host responses however did not correlate with other clinical features including the duration of cold symptoms and symptom severity (Fig. 8A). Although these pathogen-host-response correlations are a general pattern, not all samples display this trend. For example, several samples with high bacterial pathogen abundance lack a strong bacterial host response. In addition, one outlier (marked * in Fig. 8A) shows an individual with a low detected bacterial pathogen abundance but a strong bacterial host response. This could indicate an immune response to an unknown bacterial species.

In addition to the association between host-response and pathogen abundance, we also tested for host-response correlations with other clinical metadata. A weaker but significant (*r* = 0.33, *p* = 6.6 × 10^−7^) host-response pattern was detected between a subset of genes and patient symptom severity scores (Pediatric Rhinosinusitis Symptom Scale, PRSS) at the time of diagnosis. A total of 45 genes were differentially expressed as a function of PRSS, which subdivided into 2 expression clusters (Additional File 1: Fig S7). Cluster 1 was positively correlated with PRSS and includes the following genes: *METTL7B*,* MMP3*,* PRF1*,* GNLY*,* MMP1*,* FPR3*,* GIMAP6*,* OLFML2B*,* DESI1*,* IL12RB2*. Function enrichment analysis revealed that cluster 1 was associated with a response to infection (cellular defense response, natural killer cell mediated immunity, and cellular response to cytokine stimulus). Other pathways such as proteolysis and pyroptosis are also involved in innate host immune response by eliminating and degrading infected cells [54, 55].

### RNA-seq classifies patients into distinct groups with unique pathogen-host response profiles

After examining host responses to bacterial and viral infections individually, we considered how bacterial and viral relative abundance together impact host responses within patients. To investigate this, we used the RNA-seq abundance to bin samples into four groups: those with *low bacterial / low viral* pathogen abundance (*N* = 60, 27%), *high viral / low bacterial* pathogen abundance (*N* = 51, 23%), *high bacterial / low viral* pathogen abundance (*N* = 51, 23%), and *high bacterial / high viral pathogen* abundance (*N* = 59, 27%). Here, the thresholds of “high” and “low” pathogen abundance based on RNA-seq estimated levels (≥ 60th percentile) and not the presence/absence classification obtained from qRT-PCR and culture-based testing.

The four groups of patients display distinct host response signatures (Fig. 8E,F). As expected, samples with low bacterial and low viral pathogen abundance tend to have weak bacterial and antiviral responses (Fig. 8E). Samples with high viral abundance but low bacterial abundance display a strong antiviral pattern and a weak bacterial response. Samples with high bacterial pathogen abundance but low viral pathogen abundance are associated with a strong bacterial host response, and samples with high bacterial and viral pathogen abundance show both host responses. Again, there are several outliers that are exception to these general trends. The viral host response for individuals with both bacterial and viral pathogens was lower than the viral-only group (*p* = 0.01), and the bacterial host response for individuals with both bacterial and viral pathogens was not significantly different from the bacterial-only group (*p* = 0.82).

### Construction of host-response classifiers for predicting viral and bacterial infection

Lastly, we investigated whether host-response gene expression data alone could be used to predict the pathogen diagnostic results obtained from clinical testing (culture/qRT-PCR) as well as the re-classified groups (high-bacterial and high-viral) described above using RNA-seq. We compared several classifiers built from (1) cell-type enrichment scores calculated with xCell; (2) bacterial and viral host response scores calculated as the mean expression level of bacterial upDEGs (*N* = 548) and viral upDEGs (*n* = 273), (3) a random-forest model based on a reduced 10-gene bacterial signature, and 8-gene viral signature, and (4) a single gene biomarker, chosen as the top-ranked bacterial upDEG (*S100A12*) and viral upDEG (*CXCL11*) excluding non-protein-coding genes and pseudogenes. For the random forest model, repeated cross-validation was performed to estimate model accuracy (see Methods), and feature selection identified the following gene signatures: viral—*AMER1*, *MRAS*, *IFI27*, *PSME2*, *LAT*, *SLC38A5*, *MX1*, *BAK1*; bacterial—*VNN1*, *TNFRSF10D*, *SYAP1*, *HSPBAP1*, *NBPF9*, *FAM200B*, *GBE1*, *RB1CC1*, *FAM172A*, *PROK2*.

As shown in Table 3, the methods showed similar performance in predicting samples with bacterial and viral infections based on their ROC curves. Average AUCs for methods 1–4 were 0.78, 0.79, 0.81, and 0.76. The random forest classifier performed particularly well (AUC = 0.90) in predicting samples with high viral pathogen abundance calculated from RNA-seq, and lower (AUC = 0.75) in predicting virus-positive samples from qRT-PCR testing. However, this is in part due to the increased diversity of viral pathogens captured by the RNA-seq approach, which therefore showed a stronger correlation with host response.

**Table 3:** Performance (AUC) of RNA-seq host-response classifiers for predicting AS-associated bacterial and viral infections

	Clinical diagnostic (culture/qRT-PCR)		RNA-seq quantification	
Method	Bacterial +	Viral +	High bacterial	High viral
1. Cell type enrichment scores	0.76	0.75	0.76	0.84
2. Average expression of DEGs	0.77	0.80	0.78	0.80
3. Random forest classifier (10-gene bacterial signature, 8-gene viral signature)	0.81	0.75	0.79	0.90
4. Single gene classifier (bacterial—S100A12; viral—CXCL10)	0.70	0.72	0.78	0.85

Ultimately, these analyses suggest that host-response information alone may have diagnostic value in differentiating between viral and bacterial sinus infections, especially when the relative abundance (pathogen load) is high.

## Discussion

In this study, we performed metatranscriptomic analysis of 221 NP samples from children with clinically diagnosed acute sinusitis. Prior to this work, there has been a lack of research evaluating the use and applications of RNA-seq profiling in this clinical context. Our study provides several research contributions. First, it highlights the ability of RNA sequencing of clinical samples to accurately identify bacterial and viral pathogens associated with sinusitis infections and URTIs. Second, it provides an original dataset to assist with the development of future bioinformatic approaches for infectious disease profiling, including hundreds of reconstructed viral pathogen genomes contributing to ongoing pathogen genomic surveillance efforts. Third, it describes host-response signatures associated with bacterial and viral infections in sinusitis, which could serve as the basis for the development of biomarker assays to be used in future clinical workflows that optimize delivery of care.

Using RNA-seq we achieved an overall sensitivity of 87% and specificity of 81% in reproducing the clinical results for detection of three bacterial pathogens that are mostly commonly implicated in sinusitis [4]. RNA-seq also demonstrated a significant ability to detect viral pathogens that were also detected by the qRT-PCR panel (average sens/spec of 86%/92%), as well as predict viral load (Ct value). These accuracies are comparable to results obtained by previous studies using NGS for pathogen detection in NP samples [15, 32, 43].

For clinical decision making regarding antibiotic treatment, a key goal of sequencing-based approaches is to not only detect the pathogen of interest but also its antimicrobial genes, which can be especially challenging in mixed metagenomic samples. As proof of principle, we focused on beta-lactamase resistance in HFLU isolates, which represents a key clinical issue [56, 57]. As done previously for pediatric nose and ear samples [58], we used CARD [36] to identify beta-lactamases in RNA-seq data. This RNA-seq workflow was able to correctly detect beta-lactamase genes in 67% of the resistant HFLU isolates, with a specificity of 96%. Additionally, beta-lactam resistance SNPs in the *Haemophilus influenzae* PBP3 gene were also detected in several samples, which may represent an additional resistance mechanism that was detected by RNA-seq profiling but not covered by clinical AMR testing.

Finally, using a mapping-based consensus approach, we were able to reconstruct genomes of 196 viral pathogens with varying degrees of completeness. Reconstructed genomes confirmed read-based predictions and provided additional phylogenetic information. For example, phylogenetic analyses of some of these viruses (e.g., HCoV-OC43, RSV B, enterovirus D68) revealed their evolutionary relationships to related strains in the database, providing insights into their origins.

An advantage of metatranscriptomic RNA-seq over culture or qRT-PCR is the ability to perform a broad and untargeted analysis to detect any species whose genome is available in the reference database, which theoretically improves sensitivity of pathogen detection and discovery. Out of 221 pediatric sinusitis patients tested, 19 did not have any bacterial or viral pathogen detected by culture-based or qRT-PCR testing. RNA-seq identified plausible pathogens for acute sinusitis in 11 of these 19 samples including cases of influenza B and PIV1 that were missed by qRT-PCR. Not surprisingly, several new pathogenic bacteria and viruses were also detected in these samples and were verified by genome reconstruction and phylogenetics. These included two coronaviruses (NL63 and 229E), as well as the bacterium, *Chlamydia pneumoniae*. Other identified organisms included commensal organisms of the nasal microbiome and opportunistic pathogens that may or may not play a direct role in sinusitis (e.g., different species of *Moraxella* and *Corynebacterium*). Clarifying the role of these and other species in sinusitis etiology is a challenging goal for future work.

Using metatranscriptomics, we also examined transcriptional activity within three key bacterial pathogens: *Haemophilus influenzae* (HFLU), *Streptococcus pneumoniae* (SPN), and *Moraxella catarrhalis* (MCAT). Differential gene expression analysis revealed highly expressed virulence-associated genes, including *ompA* and *hfq* in HFLU, *ply* and *psaA* in SPN, and *ompR* in MCAT. These findings provide insight into pathogen-specific activity during sinusitis and underscore the utility of RNA-seq for functional microbiology in clinical settings. Notably, ribosomal genes were significantly enriched among highly expressed genes across all three species, reflecting their heightened metabolic activity during infection.

One of the most exciting aspects of this study is the identified host-response gene expression patterns associated with bacterial and viral sinusitis infectious. Since the pathogen composition of our patient cohort was complex including a large number of samples containing both bacterial and viral pathogens based on culture/qRT-PCR, we chose to simplify the initial comparison between virus-positive only samples versus bacteria-positive only samples. This enabled the detection of virus associated and bacteria associated host DEGs (“viral host response” and “bacterial host response”) that formed the basis of subsequent analyses. Remarkably, the magnitude of these host responses correlated significantly with the total abundance of bacterial or viral pathogens detected in the samples. Further cell type enrichment analysis revealed that these patterns were likely driven by changes in the abundance of key cell types such as M1 macrophages which associated with viral infections, and neutrophils which associated with bacterial infections. Importantly, this correlation between pathogen abundance and host-response magnitude was only identified for a limited subset of bacterial species (those previously identified as sinusitis pathogens, MCAT, SPN, HFLU) and respiratory viruses, and the correlation was absent when examining other species detected in the data that may reflect commensal organisms. This finding indicates that the relative abundance of specific bacterial and viral species within the nasopharynx is a determinant of the strength of the host immune response. This is consistent with immunology since the expression of host antiviral and antibacterial pathways are dependent on the levels of viral (e.g., dsRNA) and bacterial pathogen-associated molecular patterns (e.g., lipopolysaccharide) sensed by the host immune system. Previous studies have also reported a correlation between antiviral host responses in RNA-seq and viral load [59–61]. However, our study is unique by analyzing the interplay between a complex mixture of bacterial and viral pathogens and their impact on the host transcriptomic response.

Although traditional methods (culture and qRT-PCR) provided a simple classification of our samples based on detected presence/absence of a pre-defined set of pathogens, metatranscriptomic data enabled a more holistic classification based on pathogen abundance and host-response information (Fig. 8). Similar approaches have been used by previous studies such as Wesolowska-Andersen et al. [15] which stratified samples into “Virus-High” and “Virus-Low” groups based on viral read counts. When taking both pathogen abundance and host-response information into consideration, the samples could be similarly subdivided into four main groups: those with a “low” abundance of bacterial or viral pathogens which tend to lack a host-response, and those with a “high” abundance of bacterial pathogens, respiratory viruses, or both, which tend to show the expected host responses. Interestingly, the observed correlation between pathogen abundance and host-response is not perfect; there are several outlier samples which exhibited a strong host-response pattern and yet lack a detected pathogen, and other samples which contained a high pathogen abundance but lack a detectable host response. For the former category, it is possible that those samples contained other pathogens that were not included in our pathogen panel, which may include opportunistic infections by commensal organisms for example. For the latter category, these cases could indicate delayed host-responses in patients at the time of sampling, shedding of viral RNA at a post-infection time point which may be associated with a reduced host response, or simply an imperfect correlation between host responses and pathogen abundance. Nevertheless, future research focusing on host responses of patients with infectious disease and factors that account for discrepancies between detected pathogen abundance could clarify mechanistic understanding of disease etiology.

Finally, we compared a variety of methods to classify infection types and predict pathogen abundance using host-response information alone. By leveraging four distinct methods—including random-forest modeling, host-response gene signatures, cell-type enrichment via xCell, and pathogen-specific upregulated DEGs—we achieved moderate classification accuracy but with room for future improvement. Overall, a Random-forest classifier trained on bacterial and viral gene signatures demonstrated high predictive performance, particularly for samples with elevated viral loads (AUC = 0.90).

There are several limitations of our study that could account for variation in the results obtained. First, the classification into viral and bacterial infection was inferred based on the presence/absence of bacterial and viral pathogens in the nasopharynx. Although the mucosa of the nasopharynx and the sinuses are connected, pathogens in the nasopharynx and the sinus cavities could differ in important ways. Moreover, some of these organisms may be present as commensals and their presence alone does not necessitate an infection [62–65]. However, as mentioned in the background section, detection of bacterial pathogens in the nose appears to be a useful marker associated with likelihood of benefit from antibiotic treatment, regardless of whether these pathogens are truly present in the sinuses or causing an infection [6]. Second, the enrollment criteria for this study recruited patients experiencing symptoms for at least 6 days when sampled. Since peak shedding of some viruses can occur within 48 h of symptom onset, the chosen sampling time may have led to a reduced sensitivity of viral detection as well as lower coverage for genomes reconstructed. Variation in the timing of bacterial infections could also impact sensitivity of bacterial detection by RNA-seq. Third, our sensitivity for pathogen detection by RNA-seq is dependent on the depth of sequencing. Deeper sequencing may have been necessary to detect viruses, for example, that were false negatives by RNA-seq but were detected using qRT-PCR. DNA viruses in particular (e.g., adenoviruses) may have been more prone to weak detection due to the use of RNA-seq over DNA-seq. Fourth, in this manuscript, we describe associations between baseline meta-transcriptomics at other baseline variables; the potential of meta-transcriptomics to determine prognosis was not explored. Future studies that employ both metatranscriptomic and metagenomic sequencing with repeated time-series sampling of patients may overcome some of the limitations described above. Nevertheless, the current study provides a starting framework for exploring the use of high-throughput sequencing of patient samples to uncover etiology and host-response in pediatric sinusitis and other upper respiratory infections.

## Conclusions

In summary, this study applied metatranscriptomic RNA-seq to analyze 221 NP samples from children with clinically diagnosed acute sinusitis. Not only was metatranscriptomics highly accurate in its ability to detect known bacterial pathogens associated with acute sinusitis and a diverse set of clinically relevant viruses based on comparison with culture or qRT-PCR, but untargeted analysis revealed additional pathogens that are plausible causes of infection and may warrant further attention in future studies. Furthermore, the analysis of host gene expression revealed distinct host responses that differentiated bacterial from viral infections, and the magnitude of these host responses showed a significant correlation with pathogen load. Ultimately, these results reveal the potential of metatranscriptomics for dual analysis of pathogen and host-response in pediatric acute sinusitis and upper respiratory infections in general. The identified molecular signatures of bacterial and viral infections create new avenues for development of future diagnostic approaches.

## Supplementary Information

Additional file 1

Additional file 2

## Data Availability

Code and processed data associated with this paper is available at https://github.com/doxeylab/pediatric-sinusitis-transcriptome [66]. All metatranscriptomic sequencing data associated with this project has been deposited to the NCBI Sequence Read Archive under project accession PRJNA1212169. Reconstructed viral genome sequences have been deposited in FigShare [67].

## Abbreviations

ADV: Human adenovirus
AMR: Antimicrobial resistance
ANI: Average nucleotide identity
AS: Acute sinusitis
AUC: Area under the curve
Bacterial upDEGs: DEGs with increased expression in bacterial infections
CARD: Comprehensive Antibiotic Resistance Database
COV: Coronavirus
Ct: Cycle threshold associated with qRT-PCR
DEG: Differentially expressed gene
EVD68: Enterovirus D68
HFLU: Haemophilus influenzae
HRV: Human rhinovirus
INFA: Influenza A
INFB: Influenza B
INFC: Influenza C
MCAT: Moraxella catarrhalis
MPV: Human metapneumovirus
NP: Nasopharyngeal (NP)
PIV1: Parainfluenza virus 1 (PIV1)
PIV2: Parainfluenza virus 2 (PIV2)
PIV3: Parainfluenza virus 3 (PIV3)
PIV4: Parainfluenza virus 4 (PIV4)
PRSS: Pediatric Rhinosinusitis Symptom Scale
RGI: Resistance gene identifier
ROC: Receiver operator curve
RPKM: Reads per kilobase of reference sequence per million total sequencing reads
RPM: Reads per million
RSV: Respiratory syncytial virus
Sens: Sensitivity
Spec: Specificity
SPN: Streptococcus pneumoniae
URTI: Upper respiratory tract viral infection
Viral upDEGs: DEGs with increased expression in viral infections
