# Supplementary material for: Metatranscriptomic profiling reveals pathogen and host response signatures of pediatric acute sinusitis and upper respiratory infection
Source: Genome Med. 2025 Mar 17;17:22. doi: 10.1186/s13073-025-01447-3 (PMC11912616; doi:10.1186/s13073-025-01447-3)
Supplement: Supplementary file 1 — Additional file 1 [file 13073_2025_1447_MOESM1_ESM.pdf]

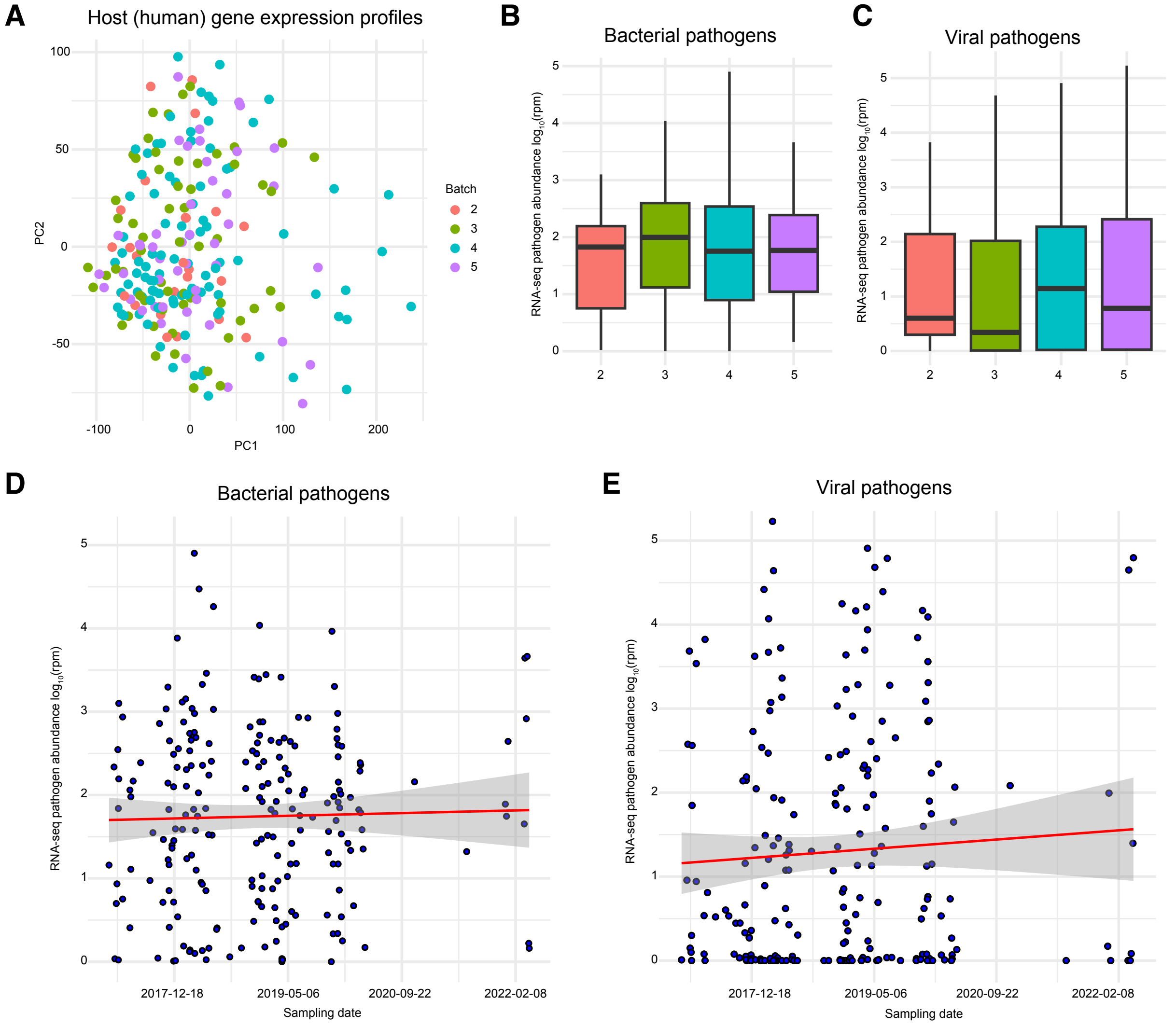

Fig S1. Investigation of potential batch effects across batches 2-5. (**A**) PCA plot of all 221 samples based on complete host (human) transcriptomic profiles. Distribution of total bacterial pathogen (**B**) and viral pathogen (**C**) abundance across batches. No significant differences were detected. Distribution of total bacterial (**D**) and viral (**E**) pathogen abundance values versus sampling date. No significant patterns were detected.

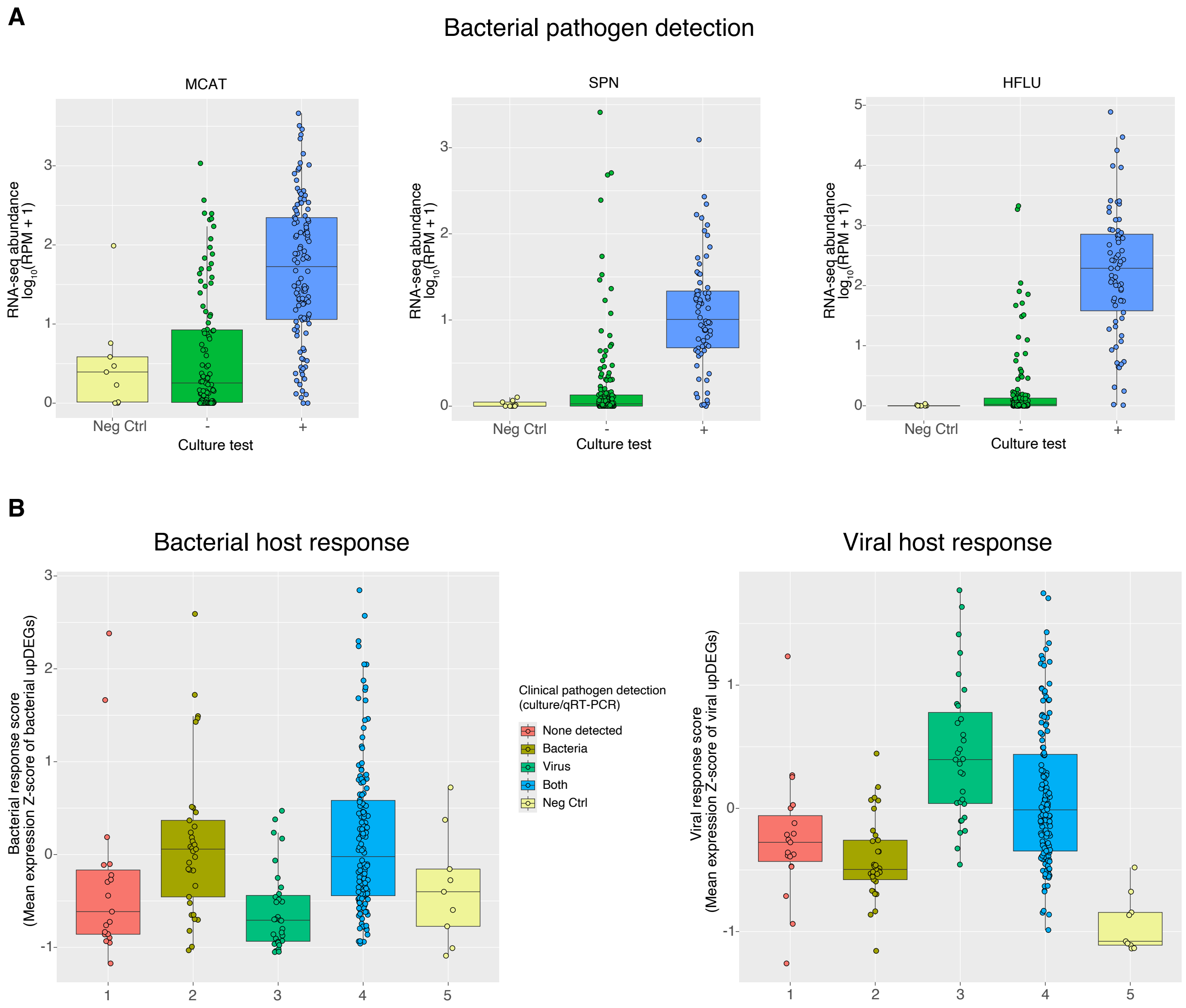

Fig S2. Bacterial pathogen abundance and host-responses detected by metatranscriptomics including nine negative control samples. (**A**) Abundance of MCAT, SPN, and HFLU in culture positive and negative samples as well as negative controls based on Kraken2 taxonomic profiling. (**B**) Bacterial and viral host responses based on average Z-scores of bacterial-associated and viral-associated upDEGs.

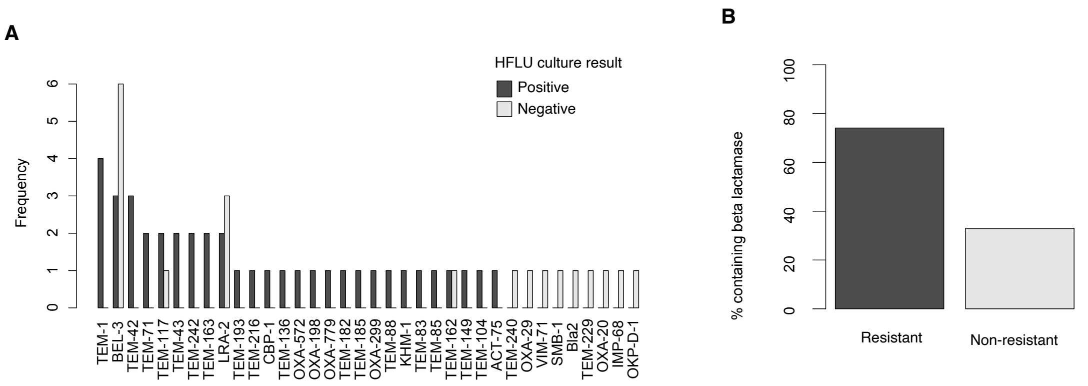

Fig S3. Detected beta-lactamase genes by CARD in resistant versus non-resistant HFLU samples. (**A**) Frequency histogram of genes detected across all HFLU-positive and negative samples (based on culture tests). (**B**) Percent of resistant and non-resistant samples with detected beta lactamase genes.

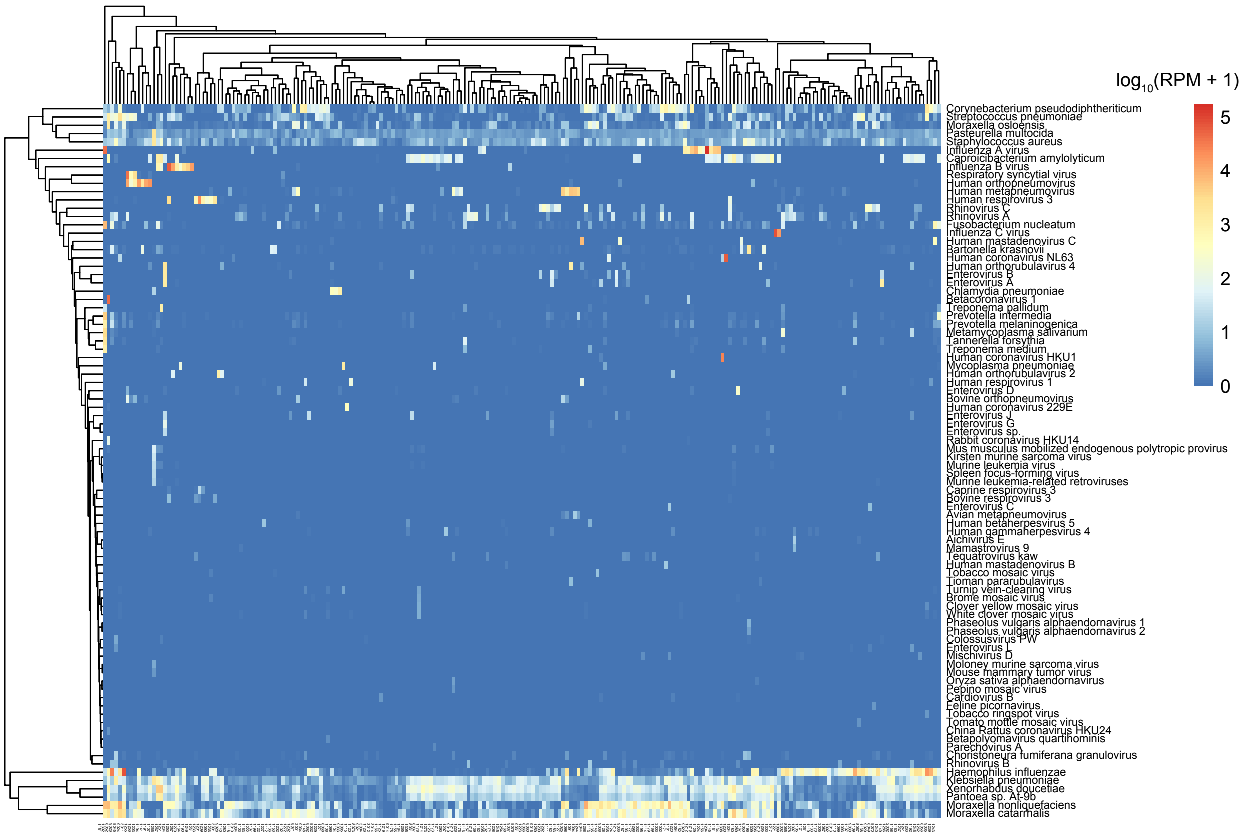

Fig S4. Abundance heatmap of microbial species in 221 patient metatranscriptomes. The heatmap displays the relative abundance of each organism in each sample as estimated by Kraken 2. Organisms with a log10(rpm) abundance greater than 3 in any sample were included, with a lower threshold (0.3) used for viruses. The heatmap was produced using pheatmap in R with samples and taxa clustered using Euclidean distances and the "complete" method.

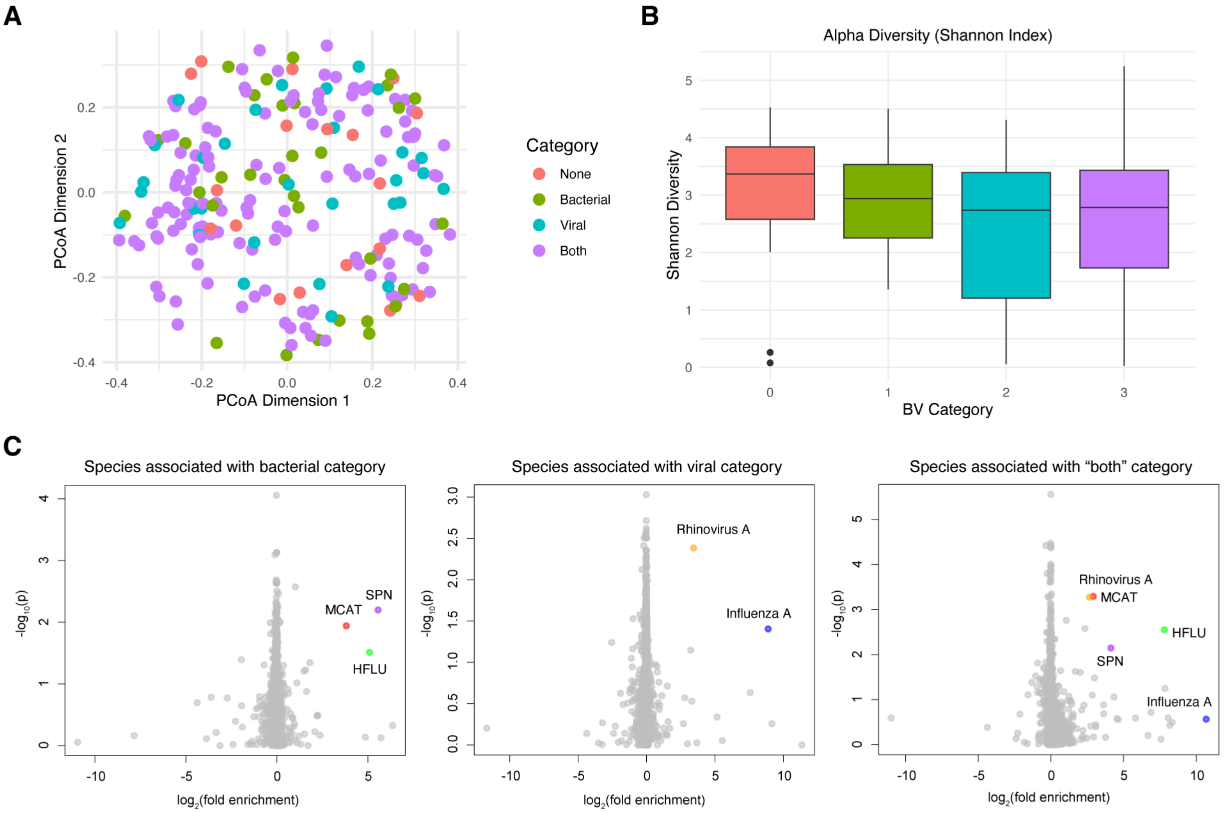

Fig S5. Microbiome analysis of 221 patient metatranscriptomes. (**A**) PCoA ordination of all samples colored by clinical category (no pathogens detected, bacterial, viral, both). (**B**) Comparison of alpha diversity (Shannon index) across the four categories. No significant differences were detected between individual groups, whereas samples associated with infections (groups 1-3) had significantly reduced average Shannon diversity compared to group 0 ($p < 0.05$). (**C**) Species associated with groups 1, 2, and 3 were identified computing the log2 fold enrichments over "group 0" as well as by abundance comparisons using Wilcoxon rank sum tests, resulting in $-\log_{10}(p$ values). Species with log2 fold changes greater than 1 and less than -1, with nominal *p*-values < 0.05 were explored. As expected, the three bacterial pathogens (MCAT, SPN, HFLU) were detected, as well as two commonly detected viruses (Influenza A and Rhinovirus A).

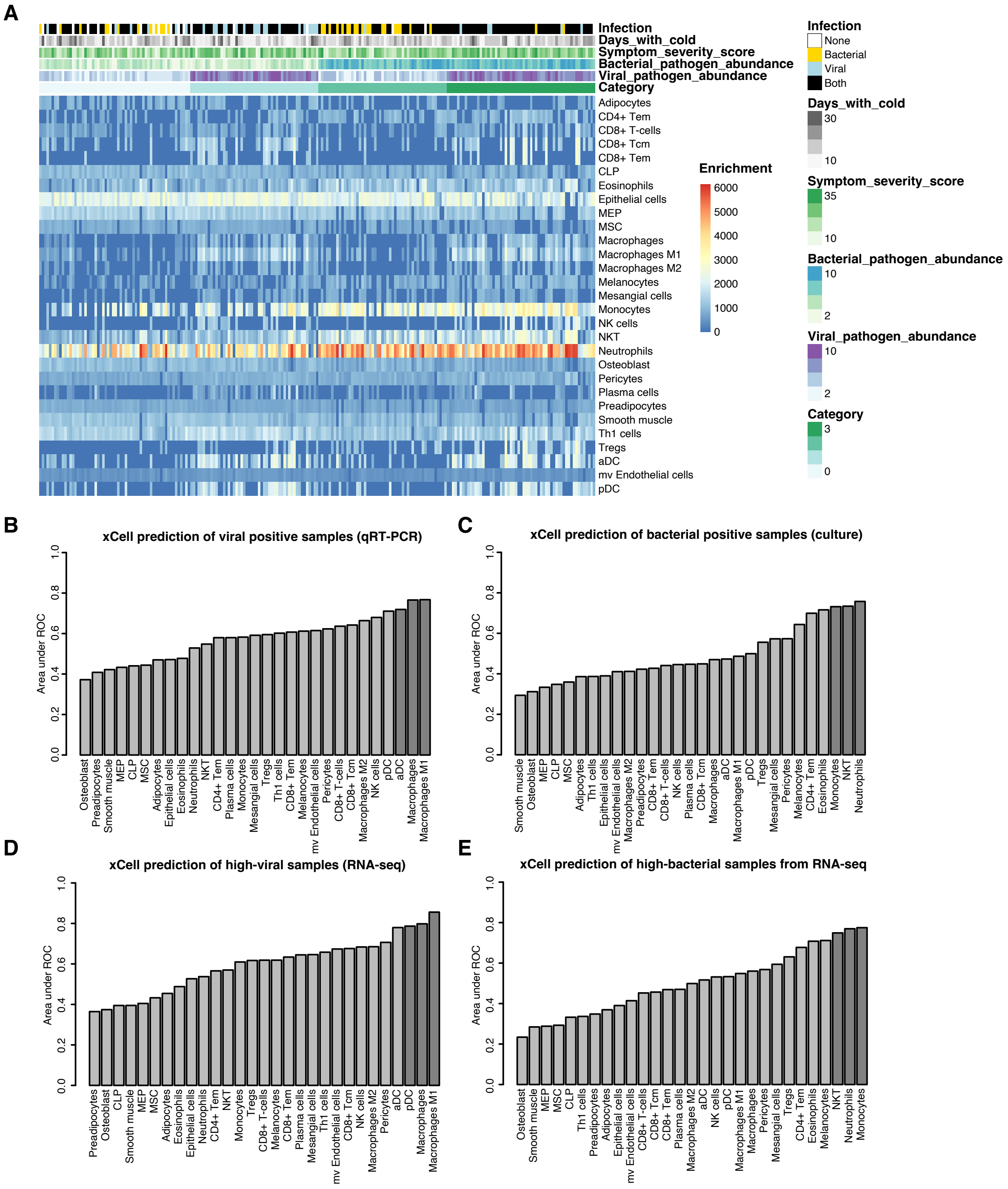

Fig S6. Cell type enrichment analysis of NP swab RNA-seq datasets from 221 patients. (**A**) Heatmap showing enrichment scores for all cell types with p-values < 0.1. (**B**-**E**) Ability of cell enrichment scores to predict bacterial and viral infections (culture/qRT-PCR categories as well as RNA-seq categories) as indicated by area under ROC curves.

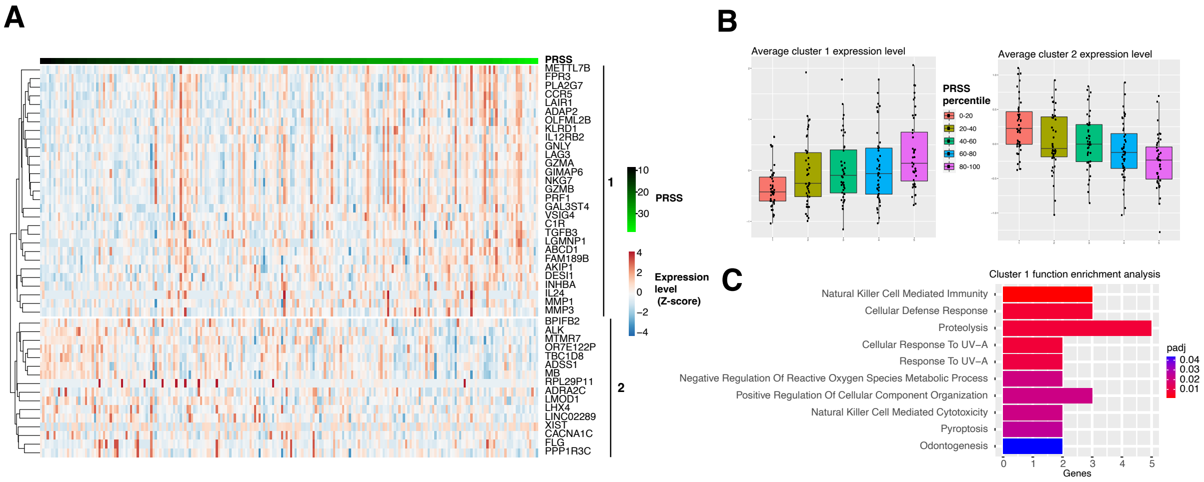

Fig S7. Differential host response expression analysis based on patients' symptom severity score (PRSS) at time of sample collection. (**A**) Heatmap displays the DEGs with $q < 0.05$. A total of 45 genes were differentially expressed and are divided into 2 clusters on the heatmap based on their expression patterns. (**B**) Average expression level (Z-scores) of genes in cluster 1 and cluster 2 for five PRSS percentile categories. (**C**) Significantly enriched ($q < 0.05$) GO Biological Process 2021 database pathways results using EnrichR of gene cluster 1.
